# Genetic and Genomic Analyses of Service Sire Effect on Female Reproductive Traits in Holstein Cattle

**DOI:** 10.3389/fgene.2021.713575

**Published:** 2021-09-03

**Authors:** Ziwei Chen, Luiz F. Brito, Hanpeng Luo, Rui Shi, Yao Chang, Lin Liu, Gang Guo, Yachun Wang

**Affiliations:** ^1^Key Laboratory of Animal Genetics, Breeding and Reproduction, MARA, National Engineering Laboratory of Animal Breeding, College of Animal Science and Technology, China Agricultural University, Beijing, China; ^2^Department of Animal Sciences, Purdue University, West Lafayette, IN, United States; ^3^Beijing Dairy Cattle Center, Beijing, China; ^4^Beijing Sunlon Livestock Development Company Limited, Beijing, China

**Keywords:** dairy cattle, genetic evaluation, genome-wide association study, paternal effect, reproductive traits, service sire

## Abstract

Fertility and reproductive performance are key drivers of dairy farm profitability. Hence, reproduction traits have been included in a large majority of worldwide dairy cattle selection indexes. The reproductive traits are lowly heritable but can be improved through direct genetic selection. However, most scientific studies and dairy cattle breeding programs have focused solely on the genetic effects of the dam (GED) on reproductive performance and, therefore, ignored the contribution of the service sire in the phenotypic outcomes. This study aimed to investigate the service sire effects on female reproductive traits in Holstein cattle from a genomic perspective. Genetic parameter estimation and genome-wide association studies (GWAS) were performed for the genetic effect of service sire (GESS) on conception rate (CR), 56-day non-return rate (NRR56), calving ease (CE), stillbirth (SB), and gestation length (GL). Our findings indicate that the additive genetic effects of both sire and dam contribute to the phenotypic variance of reproductive traits measured in females (0.0196 vs. 0.0109, 0.0237 vs. 0.0133, 0.0040 vs. 0.0289, 0.0782 vs. 0.0083, and 0.1024 vs. 0.1020 for GESS and GED heritability estimates for CR, NRR56, CE, SB, and GL, respectively), and these two genetic effects are positively correlated for SB (0.1394) and GL (0.7871). Interestingly, the breeding values for GESS on insemination success traits (CR and NRR56) are unfavorably and significantly correlated with some production, health, and type breeding values (ranging from −0.449 to 0.274), while the GESS values on calving traits (CE, SB, and GL) are usually favorably associated with those traits (ranging from −0.493 to 0.313). One hundred sixty-two significant single-nucleotide polymorphisms (SNPs) and their surrounding protein-coding genes were identified as significantly associated with GESS and GED, respectively. Six genes overlapped between GESS and GED for calving traits and 10 genes overlapped between GESS for success traits and calving traits. Our findings indicate the importance of considering the GESS when genetically evaluating the female reproductive traits in Holstein cattle.

## Introduction

In recent decades, the genetic selection for functional traits, including reproductive performance, has received great emphasis in dairy cattle selection indexes, aiming to achieve more balanced and sustainable breeding goals (Egger-Danner et al., 2015). Female fertility has only been broadly included in national selection indexes of dairy cattle breeding programs over the past five decades (Cole and VanRaden, 2018). Although most dairy cattle breeding programs focus only on the genetic effects of the dam (GED) when genetically evaluating fertility and calving traits, there are evidence that the service sire may also have a genetic influence on female reproductive performance (Barton et al., 1984; Van Tassell et al., 2003; Averill et al., 2004)—for instance, the direct effects of the service sire (e.g., semen quality and viability) on female reproductive performance have been considered as indirect effects (Jansen, 1985). Jaton et al. (2017) reported that the heritability estimates of service sire on embryo quality were lower than the donor (0.02 versus 0.04) but still statistically significant. In 2008, a national evaluation model of sire conception rate (SCR) was established in the United Sates by the Animal Improvement Program Laboratory of the United States Department of Agriculture (Norman et al., 2008). SCR is measured as confirmed pregnancy ratio (in percentage) of each service sire. They also implemented a sire–maternal grandsire (S-MGS) model to estimate the genetic component of service sire in calving performance (Van Tassell et al., 2003; Jiang et al., 2018). Even though low heritability estimates have been reported for indirect indicators of male fertility (Berry et al., 2011; Tiezzi et al., 2013), it can still be improved through genomic selection (Lillehammer et al., 2011). Hence, understanding the genetic mechanisms underlying male fertility and developing accurate genomic prediction models are of great importance but still underexplored.

The determination of the genetic effect of service sire (GESS) on reproductive traits relies on genetic and genomic analyses, including the estimation of genetic parameters and genome-wide association studies (GWAS). Previous studies have identified interesting genomic regions associated with SCR, as reviewed by Taylor et al. (2018). However, there are few reports of the GESS on other reproductive traits at the genomic level (Fang et al., 2019; Jiang et al., 2018). In this context, the main objectives of this study were as follows: (Egger-Danner et al., 2015) to investigate the genetic background of service sire on female reproductive performance, including conception rate (CR), 56-day non-return rate (NRR56), calving ease (CE), stillbirth (SB), and gestation length (GL) in Holstein cattle and (Cole and VanRaden, 2018) to identify genomic regions and candidate genes associated with GESS on female reproduction performance.

## Materials and Methods

### Phenotypic and Genomic Data

#### Phenotypes

Records of birth dates, insemination events, pregnancy diagnoses, and calving information collected in 39 farms (Sunlon Livestock Development Co. Ltd., Beijing, China) from 1987 to 2020 were extracted from the AfiFarm software (AfiFarm^1^) and used in this study. The reproductive traits include CR (1 = pregnant, 0 = non-pregnant), NRR56 (1 = non-return, 0 = return), CE (scores from 1 to 3, in which 1 = unassisted, 2 = easy pull, 3 = hard pull and surgery needed), SB (1 = calf was alive 24 h after birth and 2 = calf was dead), and GL (in days). The last insemination before a positive pregnancy diagnosis in each parity was considered as pregnancy. Furthermore, the cows with calving records but without a positive pregnancy diagnosis were considered pregnant when last inseminated before calving. Insemination records that had the next insemination within 1–17 days or had neither insemination nor calving records after that time period were excluded from the NRR56 calculation. CE and SB were directly coded from raw data after excluding ambiguous records (3%) caused by mis-recording. GL records lower than 260 and greater than 302 days were also deleted. A descriptive summary for each trait after data editing is shown in Table 1. In total, 163,818 Holstein cows had phenotypes and were serviced by 1,952 bulls. The average (±SD) number of services per bull was 489 ± 1,947. A pedigree file spanning over 13 generations was provided by the Beijing Dairy Cattle Center (BDCC, Beijing, China) and consisted of 503,118 cows and 151,273 bulls born between 1957 and 2020. The estimated breeding values (EBVs) for six production traits (milk yield, milk protein yield, milk protein ratio, milk fat yield, milk fat ratio, and somatic cell score), two health traits (reproductive diseases and udder diseases), and five type traits (dairy character, milking system, capacity, rump, and overall conformation) traits were also provided by BDCC.

**TABLE 1:** Descriptive statistics for the phenotype of reproductive traits in Holstein cattle.

Trait	Mean	*SD*	Minimum	Maximum	*N*
CR	0.43	0.49	0	1	837,655
NRR56	0.50	0.50	0	1	857,821
CE	1.06	0.27	1	3	259,042
SB	1.07	0.25	1	2	273,367
GL	278.36	6.18	260	302	258,611

*CR, conception rate (0 or 1); NRR56, 56-day non-return rate (0 or 1); CE, calving ease (1, 2, or 3); SB, stillbirth (0 or 1), GL, gestation length (day); SD, standard deviation; N, number of records.*

#### Genotypes

A total of 3,477 Holstein bulls were genotyped with the Illumina BovineSNP50 BeadChip (Illumina, Inc., San Diego, CA, United States) containing 54,609 single-nucleotide polymorphism (SNP) markers. These genotypes were imputed to the Illumina 150K Bovine Beadchip (containing 123,268 SNPs) using the BEAGLE v5.1 software (Browning et al., 2018) and a reference population consisting of 3,119 cows and 81 bulls. The SNP information was updated from an older version of the cattle reference genome (UMD 3.1) assembly to the current one (ARS-UCD 1.2) using the UCSC LiftOver tool^2^. Eight thousand five SNPs with missing position in the latest reference genome were removed from further analyses. The reference population was divided into a sub-reference population (2,725 individuals genotyped with the 150K SNP panel) and a sub-validation population (475 individuals genotyped with the 150K SNP panel but masked to only the 50K panel SNPs) for assessing the accuracy of genotype imputation as the concordance rate of imputed SNPs. Only SNPs with imputation accuracy greater than 90% were kept for further analyses. Furthermore, SNPs with minor allele frequency lower than 0.05, unknown chromosome or genome position, and extreme deviation from the Hardy–Weinberg equilibrium (*p*-value lower than 10^–6^) were removed. After data editing, 109,274 SNPs located in the autosomes and pseudo-autosomal regions of the X chromosome were retained in the dataset.

### Estimation of Genetic Parameters

The variance and covariance components for each reproductive trait were estimated using the AI-REML algorithm implemented in the DMUAI module of the DMU v6 software (Madsen et al., 2006). A previous study evaluating similar traits indicated that the heritability estimates of linear and threshold models tend to be similar (Meijering, 1985; Boichard and Manfredi, 1994). Therefore, the following linear mixed model was fitted:

$$y=X\beta +Z_{1}u_{\text{m}}+Z_{1}u_{\text{f}}+W_{1}pe_{\text{m}}+W_{2}pe_{\text{f}}+Z_{2}hym+e$$

where *y* represents the vector of phenotypic observations (i.e., CR, NRR56, CE, SB, or GL), β is the vector of fixed effects included in the model, in which different systematic effects were fitted for each trait [i.e., AI technician, parity, semen type, and number of inseminations for CR and NRR56 and calf sex, parity, and group of calf size (divided according to their birth weights: 30–40, 40–50, and 50–60 kg) for CE, SB, and GL]. All of the fixed effects significantly (*P* < 0.05) influenced the dependent variables (CR, NRR56, CE, SB, and SB) and were identified based on mixed model analysis using the PROC MIXED function implemented in the SAS software (version 9.1.3; SAS Institute Inc., Cary, NC, United States); *u*_m_ and *u*_f_ are the vectors of the random animal effects accounted by GESS and GED, respectively; *pe*_m_ and *pe*_f_ are the vectors of the random permanent environmental effects of the service sire and dam, respectively; *hym* is the vector of the random herd–year–month effects; *e* is the vector of the random residual effects; and, *X*, *Z*_1_, *W*_1_, *W*_2_ and *Z*_2_ are the corresponding incidence matrices. We assumed that:

$$\left(\begin{array}{c} u_{\text{m}}\\ u_{\text{f}}\\ pe_{\text{m}}\\ \begin{array}{c} pe_{\text{f}}\\ \begin{array}{c} h\\ e \end{array} \end{array} \end{array}\right)∼N\left(O,V\right)$$

with:

$$V=\left(\begin{array}{ccccc} A⊗\left(\begin{array}{cc} \sigma_{\text{m}}^{2} & \sigma_{m,f}\\ \sigma_{m,f} & \sigma_{\text{f}}^{2} \end{array}\right) & 0 & 0 & 0 & 0\\ 0 & I⊗\sigma_{\mathrm{pef}}^{2} & 0 & 0 & 0\\ 0 & 0 & I⊗\sigma_{\mathrm{pem}}^{2} & 0 & 0\\ 0 & 0 & 0 & I⊗\sigma_{\mathrm{hym}}^{2} & 0\\ 0 & 0 & 0 & 0 & I⊗\sigma_{\text{e}}^{2} \end{array}\right)$$

where $\sigma_{\text{m}}^{2}$ and $\sigma_{\text{f}}^{2}$ are the additive genetic variances of service sire and dam, respectively; σ_m,f_ is the genetic covariance of service sire and dam; $\sigma_{\text{pem}}^{2}$ and $\sigma_{\text{pef}}^{2}$ are the permanent environmental variances of service sire and dam, respectively;$\sigma_{\text{hym}}^{2}$ is the herd–year–month variance; $\sigma_{\text{e}}^{2}$ is the residual variance; ⊗ is the Kronecker product function; *A* is the additive genetic relationship matrix among the animals; and *I* is an identity matrix. For calving traits, the model could be considered as an improved sire–dam model that assumes that the GESS has a genetic covariance with the GED. Compared to the traditional evaluation models of calving traits that only consider the animal effect, the current model includes both service sire and dam effects in the female reproductive performance phenotypes through GESS and GED, respectively. Differently from direct and maternal (paternal) effects usually assumed to be correlated, this was not the case for the genetic components of service sire and dam in the current study. Therefore, the GESS heritability estimates were calculated as follows:

$$\begin{array}{c} h_{\text{m}}^{2}\\ =\sigma_{\text{m}}^{2}/\left(\sigma_{\text{m}}^{2}+\sigma_{\text{f}}^{2}+2\times \sigma_{m,f}+\sigma_{\text{pem}}^{2}+\sigma_{\text{pef}}^{2}+\sigma_{\text{hym}}^{2}+\sigma_{\text{e}}^{2}\right) \end{array}$$

and the repeatability estimates were calculated as follows:

$$\begin{array}{c} re_{\text{m}}=\left(\sigma_{\text{m}}^{2}+\sigma_{\text{pem}}^{2}\right)/(\sigma_{\text{m}}^{2}+\sigma_{\text{f}}^{2}+2\times \sigma_{m,f}+\sigma_{\text{pem}}^{2}\\ \;+\sigma_{\text{pef}}^{2}+\sigma_{\text{hym}}^{2}+\sigma_{\text{e}}^{2}) \end{array}$$

The GED repeatability was estimated in the same way but replacing $\sigma_{\text{m}}^{2}\mathrm{and}\sigma_{\text{pem}}^{2}$ in the numerator by $\sigma_{\text{f}}^{2}+\sigma_{\text{pef}}^{2}$.

The standard error of the heritability and repeatability estimates, respectively, were calculated using the Delta method (Su et al., 2007). A Wald test was carried out to determine the statistical difference between the genetic parameter estimates and zero. In addition, correlations between the genomic breeding values for GESS of the reproduction traits, as well as production, health, and type traits, were estimated using the method proposed by Calo et al. (1973) and bull EBVs (filtered based on EBV reliability, as described below). Standard errors (SE) of the approximate genetic correlations were calculated based on the formula proposed by Sokal and Rohlf (1981). The predicted transmitting ability (PTA) of six production traits (milk yield, milk protein yield, milk protein ratio, milk fat yield, milk fat ratio, and somatic cell score), two health traits (reproductive disease and udder disease), and five type traits (dairy character, milking system, capacity, rump, and overall conformation) with reliabilities higher than 20, 20, and 30%, respectively, were provided by BDCC. The statistical models used for the genetic evaluation of these traits are described in Miglior et al. (2009) and Wu et al. (2013). Individuals with PTA reliabilities for GESS on reproductive traits above 40% were used for the calculation of correlation of breeding values.

### Genome-Wide Association Study and Functional Enrichment Analyses

The “Fixed and random model Circulating Probability Unification” (FarmCPU) R package (Liu X. et al., 2016) was used to perform single-SNP regression analyses. FarmCPU is a multi-locus model that incorporates multiple markers simultaneously as covariates to partially remove the confounding effect between testing markers and kinship (Liu X. et al., 2016). De-regressed proofs (DRP) of the GESS and GED for female reproductive traits were derived following the procedures suggested by Wiggans et al. (2011). Individuals with accuracies greater than 10% were used as dependent variables in the GWAS model. The obtained *p*-values were corrected for multiple testing by calculating the false discovery rate (FDR) (Benjamini et al., 2001) at the 5% genome-wise level. Quantile–quantile (Q–Q) plots and the inflation factor λ (Devlin and Roeder, 1999) were used to investigate population stratification by comparing the observed and expected distributions of –log(*p*-value).

Positional genes located at up to 200 kb upstream and downstream of the significant SNPs were identified using the BiomaRt package (Durinck et al., 2005). This 200-kb window was defined based on the linkage disequilibrium level of the studied population (Supplementary Figure 1). The ClueGO module in Cytoscape (Bindea et al., 2009) was used to identify candidate genes, Gene Ontology (GO), and Kyoto Encyclopedia of Genes and Genomes (KEGG) terms. Furthermore, the Cattle Quantitative Trait Locus (QTL) database (Cattle QTLdb Release 42^3^) was used to identify important trait–QTL associations previously reported in the literature.

## Results and Discussion

### Descriptive Statistics of Phenotype

The descriptive statistics of the five reproductive traits evaluated are shown in Table 1. For the insemination success traits (CR and NRR56), the mean CR was 43 ± 49% and the mean NRR56 was 50 ± 50% in Chinese Holstein cattle. These estimates are lower than those reported by Guo et al. (2014), which were inferred from the number of services recorded from 2001 to 2010. The average CE, SB, and GL were 1.06 ± 0.27, 1.07 ± 0.25, and 278.36 ± 6.18 days, respectively. The proportion of CE scores higher than 1 was 5.21%, and the SB rate was 6.67%. Vieira-Neto et al. (2017) reported a mean GL of 276 ± 6 days in Holstein cows, which is slightly lower than that of the current study. GL is affected by various environmental effects, such as temperature (McClintock et al., 2003) and cow parity number (King et al., 1985), which may have led to the discrepancy of GL between different populations.

### Genetic Parameters for Female Reproductive Traits Considering GESS

#### Heritability and Repeatability Estimates

The (co)variance components, heritability, and genetic correlations for GESS and GED for all five reproduction traits are shown in Table 2. Overall, the heritability estimates for the success traits were low, but the heritability of GESS were significantly higher than those of GED (0.020 ± 0.004 vs. 0.011 ± 0.000 for CR and 0.024 ± 0.005 vs. 0.013 ± 0.001 for NRR56), indicating that service sires actually have greater genetic impact on insemination success than the mating cows. Fertility traits are known to have low heritability (VanRaden et al., 2004), which makes it more difficult to obtain faster genetic progress compared to more heritable traits. Therefore, larger datasets and novel phenotypes (Fleming et al., 2019) should be generated for increasing genetic progress for fertility performance. The small, but significant, genetic contribution of service sires on success traits indicates the possibility of genetically improving the GESS. The repeatability estimates of these reproduction traits were almost equal to their heritabilities, except for the GED on NRR56 (0.031 ± 0.001 vs. 0.013 ± 0.001), indicating the inconsistency among these records. Hence, more repeated records are needed for greater EBV accuracies. Another reason for the low repeatability might be the ignorance of non-additive genetic effects, which could lead to the underestimation of genetic parameters. For most traits, the genetic variance of the GESS was comparable to that of the GED, demonstrating that service sires have considerable genetic contribution to female reproductive outcomes. The heritability estimates for female CR in other studies range from 0.01 to 0.03 (Azzam et al., 1988; Bagnato and Oltenacu, 1993; Muuttoranta et al., 2019). These low estimates support the estimates of GED on CR in this study. The heritability of GED on NRR56 was consistent with the value (0.01) reported by Sun and Su (2010) but different from that of Hoekstra et al. (1994) (0.04) and Eghbalsaied (2011) (0.002–0.003). These discrepancies may be attributed to the differences in the populations evaluated and the statistical models used since GESS was only included in the current study. Some studies have considered the GESS as a non-genetic random effect apart from the GED, and the heritabilities obtained from such models were shown to be lower than 0.02 for CR and NRR56 (Weigel and Rekaya, 2000; Kuhn and Hutchison, 2008). However, Tiezzi et al. (2011) suggested that the heritabilities generated using the approach mentioned above are lower than those fitting GESS. Furthermore, Tiezzi et al. (2013) reported that the heritability and repeatability of the GESS on NRR56 were both 0.01 for Italian Brown Swiss cattle. The characteristics of the two populations evaluated might have caused the discrepancy in the estimates observed. In the study of Tiezzi et al. (2013), a smaller and older population than that in the current study was used, and the cattle in their population performed better in NRR56 (0.70 for average), indicating a better management and genetic level of their herds.

**TABLE 2:** Genetic components and parameters for reproductive traits in Holstein cattle.

Parameter	CR	NRR56	CE	SB	GL
$\sigma_{ss}^{2}$	4.50 × 10^–3^	5.41 × 10^–3^	3.06 × 10^–4^	1.96 × 10^–3^	3.9844
σ_*ss*,*d*_	1.47 × 10^–4^	−3.80 × 10^–4^	8.16 × 10^–5^	8.90 × 10^–5^	3.1303
$\sigma_{d}^{2}$	2.49 × 10^–3^	3.03 × 10^–3^	2.24 × 10^–3^	2.08 × 10^–4^	3.9699
$\sigma_{pess}^{2}$	4.13 × 10^–8^	2.68 × 10^–4^	2.22 × 10^–9^	1.76 × 10^–7^	1.16 × 10^–6^
$\sigma_{ped}^{2}$	2.75 × 10^–7^	4.09 × 10^–3^	4.65 × 10^–7^	3.09 × 10^–4^	0.5436
$\sigma_{hys}^{2}$	0.0126	0.0152	0.0228	8.59 × 10^–3^	3.2778
$\sigma_{e}^{2}$	0.2099	0.2003	0.0521	0.0140	27.1262
$h_{ss}^{2}$	0.0196	0.0237	0.0040	0.0782	0.1024
$SE_{h_{ss}^{2}}$	4.10 × 10^–3^	4.50 × 10^–3^	0.0013	0.0101	8.70 × 10^–3^
*re*_*ss*_	0.0196	0.0249	0.0040	0.0782	0.1024
*SE*_*re_ss_*_	5.30 × 10^–3^	0.0058	0.0017	0.0131	0.0109
$h_{d}^{2}$	0.0109	0.0133	0.0289	0.0083	0.1020
$SE_{h_{d}^{2}}$	8.00 × 10^–4^	9.00 × 10^–4^	0.0019	9.00 × 10^–4^	3.40 × 10^–3^
*re*_*d*_	0.0109	0.0312	0.0289	0.0207	0.1160
*SE*_*re*_*d*__	1.10 × 10^–3^	0.0012	0.0029	1.80 × 10^–3^	0.0043
*r*	0.0438	−0.0937	0.0986	0.1394	0.7871
*SE*_*r*_	0.0662	0.0627	0.0811	0.0705	0.0402

*$\sigma_{ss}^{2},$ additive genetic variance of service sire; σ_ss,d_, genetic covariance of service sire and dam; $\sigma_{d}^{2},$ additive genetic variance of dam; $\sigma_{pess}^{2},$ permanent environment variance of service sire; $\sigma_{ped}^{2},$ permanent environment variance of dam;$\sigma_{hym}^{2},$ herd-year-month variance; $\sigma_{e}^{2},$ residual variance; $h_{ss}^{2},$ heritability of service sire; $SE_{h_{ss}^{2}},$ standard error of heritability of service sire; re_ss_, repeatability of service sire; SE_re_ss__, standard error of repeatability of service sire; $h_{d}^{2},$ heritability of dam; $SE_{h_{d}^{2}},$ standard error of heritability of dam; re_d_, repeatability of dam; SE_re_d__, standard error of repeatability of dam; r, genetic correlation between genetic effect of service sire (GESS) and genetic effects of the dam (GED); SE_r_, standard error of genetic correlation between GESS and GED; CR, conception rate; NRR56, 56-day non-return rate; CE, calving ease; SB, stillbirth; GL, gestation length.*

For calving traits, the heritability estimates of GESS are lower than the GED for CE (0.004 ± 0.001 vs. 0.029 ± 0.002) and similar for GL (0.102 ± 0.001 vs. 0.102 ± 0.000) but higher for SB (0.078 ± 0.010 vs. 0.008 ± 0.001). Both GESS and GED show low heritability for SB and CE, though a moderate estimation was observed for GL. Except for the GED for SB (0.021 ± 0.002) and GL (0.116 ± 0.004), the repeatabilities were almost equal to the corresponding heritabilities. Each cow has only one calving record in each parity, which results in poor consistency among repeated data. The low heritability estimates of SB and CE indicate that these traits might benefit more from genomic information. A S-MGS model was used to evaluate calving traits in previous studies, which resulted in low heritability for CE and SB (0.07–0.13) (Van Tassell et al., 2003; Heringstad et al., 2007). The S-MGS model converted the direct and maternal variances to sire and maternal grandsire variances, which was, to some extent, different from those of the present study. Due to the differences in the model used in the current study, here we simply compare our findings with the results of the S-MGS model from previous studies. The maternal heritabilities estimated by other models ranged from 0.01 to 0.13 for GL (Jamrozik et al., 2005; Crews, 2006; Mujibi and Crews, 2009) and 0.02–0.08 for CE and SB (Luo et al., 2002; Jamrozik et al., 2005; Eaglen et al., 2013). The GED variance obtained in the current study for SB was 0.0002, which is much lower than the estimates from a previous study with Norwegian Red cows (Heringstad et al., 2007). The heritability of the GED was higher than that of the GESS for CE, which may have been caused by a larger maternal effect (Jamrozik et al., 2005)—for instance, cow body conformation is genetically associated with calving difficulty (Dadati et al., 1985; Ga et al., 2021), indicating the crucial role of GED on CE.

#### Genetic Correlations

Former genomic analyses reported that some genes (e.g., *SPP1*) regulate both tissue and embryonic growth (Weintraub et al., 2004; Rangaswami et al., 2006), which is important for both the male and female aspects of calving traits. Furthermore, some genes related to spermatogenesis have been shown to affect cow reproduction performance (Peddinti et al., 2010; Li et al., 2012b; Buzanskas et al., 2017). Thus, the genetic covariances of male and female were considered. The genetic correlations between the GESS and GED were significantly different from zero for SB and GL (*P* < 0.05; based on a *t*-test). The correlation coefficient was considerably high in GL, whereas that in CE was low and non-significant (*P* > 0.05; CE: 0.099 ± 0.081; SB: 0.139 ± 0.071; GL: 0.787 ± 0.040). The results indicate the homogeneity between the GESS and the GED for SB and GL. The genetic covariances of the insemination traits between the effects of service bull and dams were usually ignored in previous studies (Berry et al., 2011). We evaluated these covariances because there are evidence of low but statistically significant correlations between GESS and GED [e.g., NRR56: 0.010 ± 0.002; (Tiezzi et al., 2013)]. However, there was no significant correlation between the two terms in the current study (CR: 0.044 ± 0.066 and NRR56: −0.094 ± 0.063). The genetic correlations between the direct and maternal effects for SB and CE can be quite variable, ranging from −0.24 to 0.12 (Luo et al., 1999; Steinbock et al., 2003; Wiggans et al., 2003; Cole et al., 2007; Heringstad et al., 2007; Vanderick et al., 2014). For GL, the correlations are usually negative and stronger (−0.13 ∼ −0.85) (Cubas et al., 1991; Bennett and Gregory, 2001; Hansen et al., 2004; Crews, 2006; Cervantes et al., 2010). These discrepancies are reasonable, because we considered both the direct effect of dam and maternal effect as dam effect.

The correlations of breeding values of the GESS of reproductive traits as well as production, health, and type traits are shown in Table 3. For all the traits, the genetic information of approximately 400–500 individuals with reliability greater than 40% for reproductive traits and 20% for the other traits was used for the calculation of breeding value correlations. Interestingly, the GESS was negatively correlated with most production and type traits (e.g., milk yield and overall conformation), while positive correlations were observed between GESS and health traits such as reproductive disorders. We found that the GESS on CR was unfavorably and significantly related to milk yield (−0.218 ± 0.039), indicating that selection exclusively on milk production might indirectly result in a decline of insemination performance of the service sire. Murray et al. (1977) reported a negative correlation between male fertility and milk yield (−0.26), which is in contrast to the positive correlation (0.13–0.29) reported by Raheja et al. (1989). Further verification about the biological correlation between male fertility and milk production is needed due to the inconsistent relationships observed. The genetic correlations between GESS on success traits and reproductive disease were positive (0.174 ± 0.043 for CR and 0.203 ± 0.042 for NRR56). Progesterone is regarded as a responsible factor for cattle ovarian follicular cysts (Silvia et al., 2002). Ramal-Sanchez et al. (2020) also suggested that progesterone is related to sperm release, which affects the fertilization ability of spermatozoa. Therefore, reproduction-related hormones might account for the potential biological relation between GESS on success traits and reproductive disease. Cows with a higher incidence of ovarian cysts tend to have lower fertility. The genetic correlation between GESS on GL and overall conformation was negative, indicating that undesirable body conformation might lead to longer GL, with worse development after birth (Bourdon and Brinks, 1982). These correlations indicate that direct selection for production, health, and type traits may have a favorable effect on service sire calving performance but may lead to an unfavorable decline in service sire mating performance.

**TABLE 3:** The correlations of breeding values for the genetic effects of service sire of reproductive traits and production, health, and type traits in Holstein cattle.

Trait^a^		CR	NRR56	CE	SB	GL
Production	Milk yield	−0.218 (0.039)	−0.334 (0.036)	−0.044 (0.034)	−0.191 (0.058)	−0.277 (0.031)
	Milk protein yield	−0.262 (0.038)	−0.402 (0.035)	−0.035 (0.034)	−0.254 (0.057)	−0.348 (0.030)
	Milk protein ratio^b^	−0.150 (0.039)	−0.188 (0.037)	0.041 (0.034)	−0.199 (0.058)	−0.250 (0.031)
	Milk fat yield	−0.277 (0.041)	−0.449 (0.037)	−0.055 (0.039)	−0.256 (0.058)	−0.300 (0.034)
	Milk fat ratio^b^	−0.048 (0.043)	−0.132 (0.041)	0.005 (0.039)	−0.135 (0.060)	0.004 (0.035)
	Somatic cell score	−0.020 (0.049)	0.152 (0.048)	0.059 (0.047)	0.313 (0.060)	0.086 (0.042)
Health	Reproductive disease	0.174 (0.043)	0.203 (0.042)	0.067 (0.042)	0.219 (0.054)	0.127 (0.037)
	Udder disease	0.188 (0.043)	0.274 (0.041)	−0.124 (0.041)	−0.004 (0.055)	0.277 (0.036)
Type	Dairy character	−0.006 (0.055)	−0.126 (0.054)	−0.003 (0.053)	−0.022 (0.065)	−0.067 (0.048)
	Milking system	−0.076 (0.047)	−0.334 (0.043)	0.130 (0.044)	−0.003 (0.059)	−0.399 (0.038)
	Capacity	0.038 (0.046)	−0.075 (0.045)	−0.061 (0.044)	−0.043 (0.059)	−0.262 (0.039)
	Rump	−0.099 (0.047)	−0.283 (0.045)	−0.001 (0.046)	−0.155 (0.059)	−0.493 (0.036)
	Overall conformation	−0.013 (0.048)	−0.236 (0.046)	0.093 (0.046)	−0.018 (0.060)	−0.386 (0.039)

*CR, conception rate; NRR56, 56-day non-return rate; CE, calving ease; SB, stillbirth; GL, gestation length. ^a^The EBVs of 400–500 individuals with reliability greater than 40% for reproductive traits and 20% for other traits were used for calculating the approximate correlations. ^b^The EBV of milk fat ratio and milk protein ratio, respectively, come from the formulas below: $EBV_{fat_{ratio}}=\frac{EBV_{fat_{yield}}\times 100-EBV_{milk_{yield}}\times fat_{ratio}}{EBV_{milk_{yield}}+milk_{yield}}$$EBV_{protein_{ratio}}=\frac{EBV_{protein_{yield}}\times 100-EBV_{milk_{yield}}\times protein_{ratio}}{EBV_{milk_{yield}}+milk_{yield}}$ where EBV_fat_ratio__, EBV_fat_yield__, EBV_milk_yield__, EBV_protein_ratio__, and EBV_protein_yield__ represent the EBV of milk fat ratio, milk fat yield, milk yield, milk protein ratio, and milk protein yield, respectively; fat_ratio_, protein_ratio_, and milk_yield_ represent the average milk fat ratio, milk protein ratio, and milk yield of cows in their second lactation.*

#### Genome-Wide Association Studies

**FIGURE 1:**
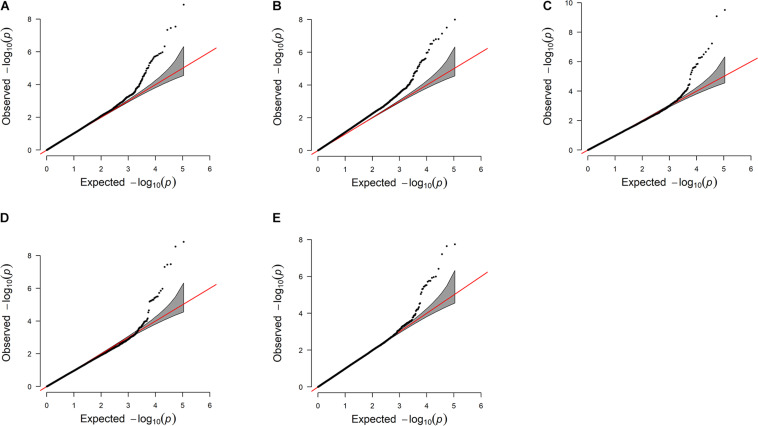
Quantile–quantile plots of the genome-wide association studies for genetic effect of service sire on reproductive traits **(A–E)**. The *x*-axis and the *y*-axis represent the expected and observed −log_10_(*P*−*value*), respectively. **(A)** Conception rate—the λ value is 1.06. **(B)** 56-day non-return rate—the λ value is 1.15. **(C)** Calving ease—the λ value is 0.96. **(D)** Stillbirth—the λ value is 0.95. **(E)** Gestation length—the λ value is 0.99.

**FIGURE 2:**
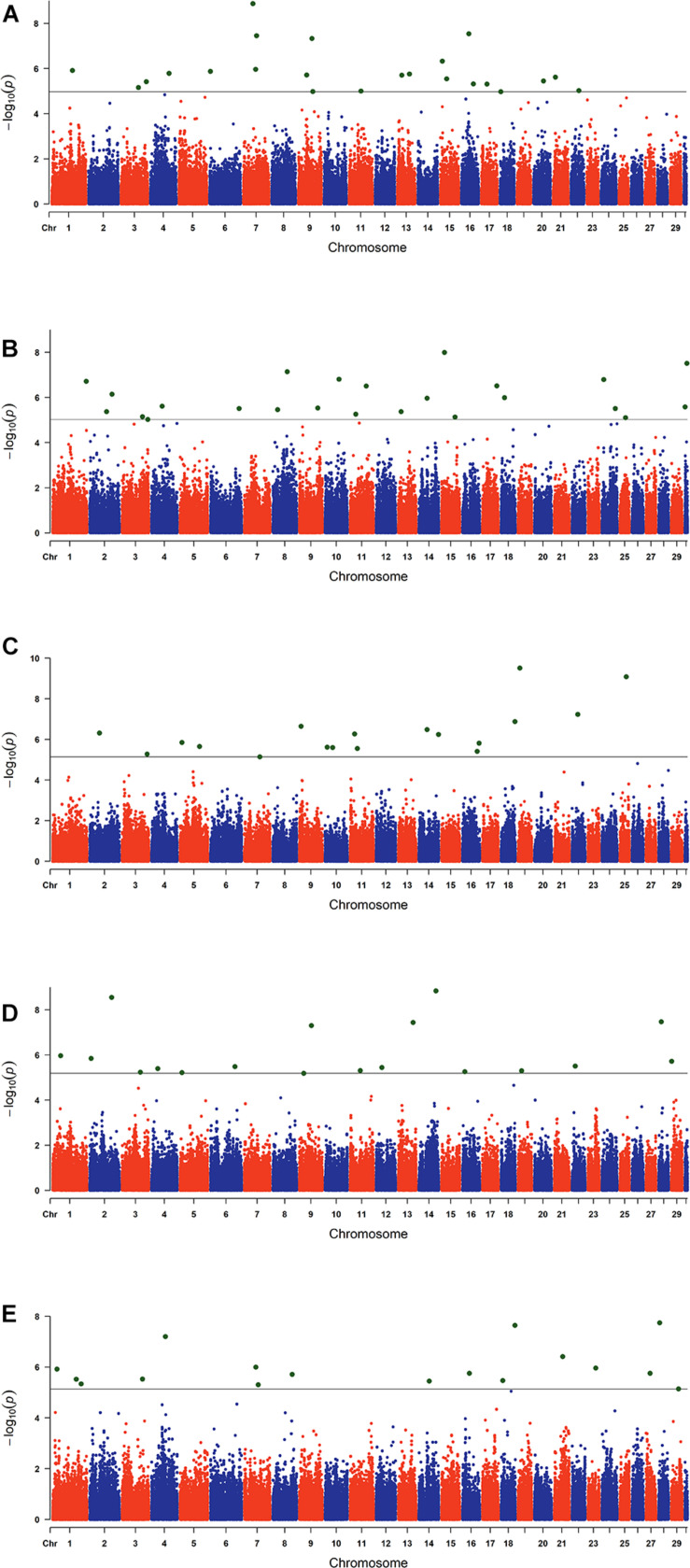
Manhattan plots of the genome-wide association studies for genetic effect of service sire on reproductive traits. The *x*-axis and the *y*-axis represent the chromosome number and the observed −log_10_(*P*−*value*), respectively. The single-nucleotide polymorphisms were plotted against their genomic positions. The lines in the plots indicate the thresholds of false discovery rate (0.05) in the corresponding traits: **(A)** conception rate, **(B)** 56-day non-return rate, **(C)** calving ease, **(D)** stillbirth, and **(E)** gestation length.

**FIGURE 3:**
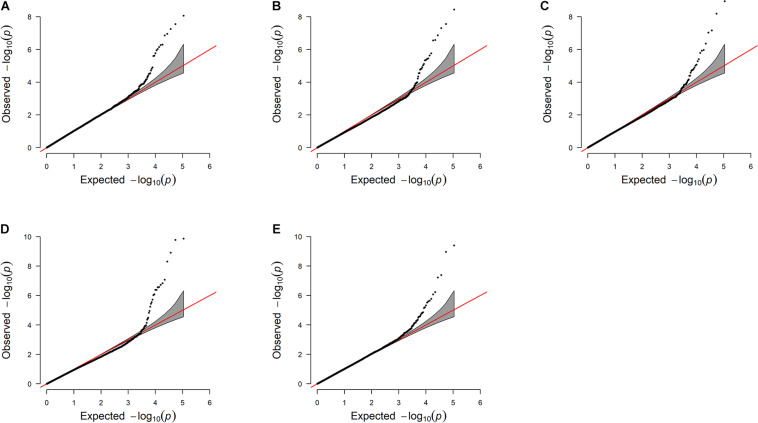
Quantile–quantile plots of genome-wide association studies for genetic effects of the dam on reproductive traits **(A–E)**. The *x*-axis and the *y*-axis represent the expected and observed −log_10_(*P*−*value*). **(A)** Conception rate—the λ value is 1.03. **(B)** 56-day non-return rate—the λ value is 0.92. **(C)** Calving ease—the λ value is 0.96. **(D)** Stillbirth—the λ value is 0.94. **(E)** Gestation length—the λ value 1.00.

**FIGURE 4:**
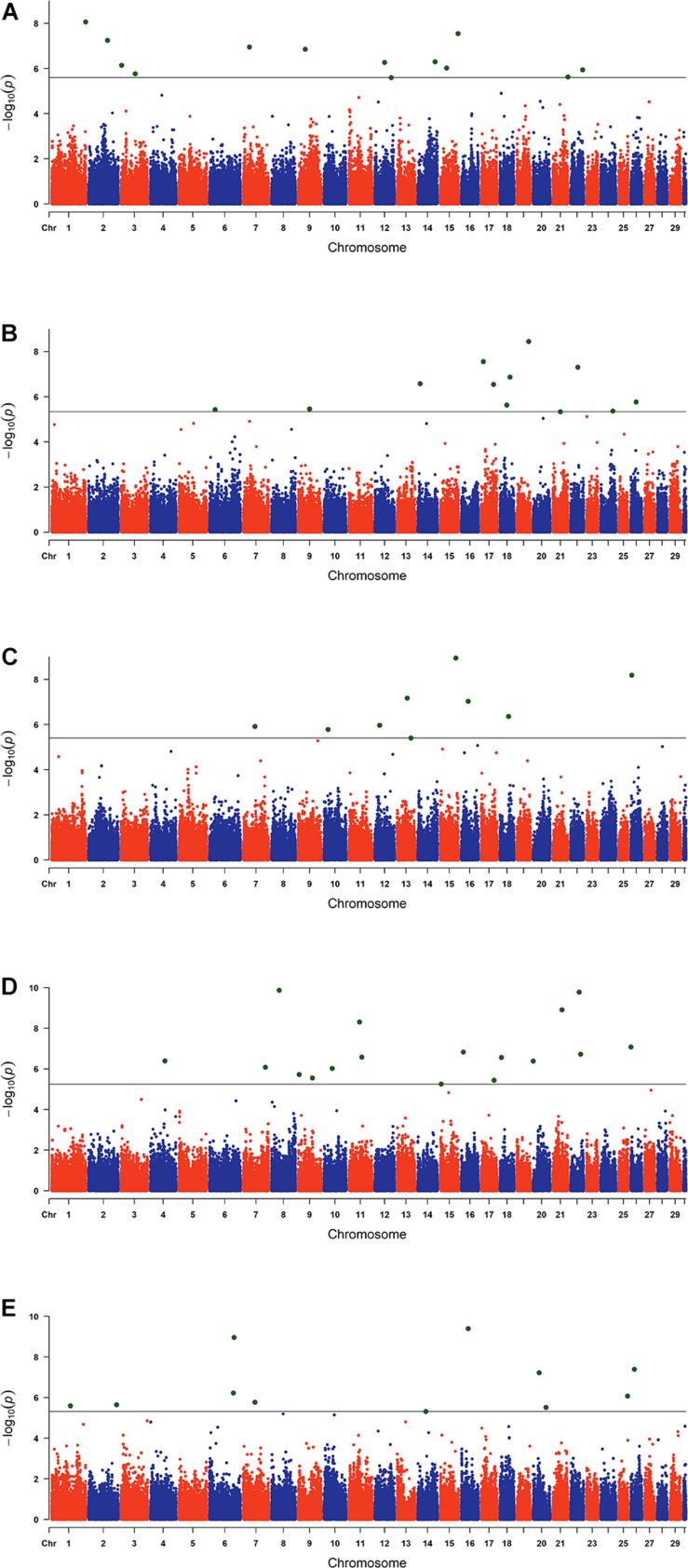
Manhattan plots of the genome-wide association studies for the genetic effects of the dam on reproductive traits. The *x*-axis and the *y*-axis represent the chromosome number and the observed −log_10_(*P*−*value*), respectively. The single-nucleotide polymorphisms were plotted against their genomic positions. The lines in the plots indicate the thresholds of false discovery rate (0.05) in the corresponding traits: **(A)** conception rate, **(B)** 56-day non-return rate, **(C)** calving ease, **(D)** stillbirth, and **(E)** gestation length.

The estimated breeding values obtained from former genetic estimation with accuracy above 10% were used for DRP calculation. Hence, the EBV of 2,996 and 1,147 individuals were used in GWAS for GESS and GED, respectively. The detailed information of the genomic regions and candidate genes for GESS and GED for the five reproductive traits are summarized in Tables 4, 5. In addition, the Q–Q plots and Manhattan plots are provided in Figures 1–4. The Q–Q plots and λ of GESS on 56-day non-return rate indicated a slight inflation of the results. A total of 162 significant SNPs were detected, including two significant SNPs located in the X chromosome (pseudo-autosomal region) (Johnson et al., 2019). The *P*-values ranged from 1.34 × 10^–10^ to 1.05 × 10^–5^, and FDR ranged from <0.001 to 0.050. Furthermore, we mapped the significant SNPs to the Cattle QTL database (see Text Footnote 3), and the overlapped QTL regions are listed in Tables 6, 7.

**TABLE 4:** Significant single-nucleotide polymorphism (SNP) and nearby genes of the genetic effects of service sire on five reproductive traits carried out by genome-wide association studies in Holstein cattle.

Trait	SNP	BTA	Position (bp)	*P*-value	MAF	Effect	FDR	Genes^a^
CR	rs29011049	1	97,448,665	1.21 × 10^–6^	0.38	0.006	0.018	***LRRC34***, *ACTRT3*, *MYNN*, *PHC3, SAMD7*, *SEC62*, *GPR160*
	rs133215257	3	79,272,169	6.96 × 10^–6^	0.26	−0.006	0.040	*PDE4B*, *MGC137454*
	rs43370565	3	115,520,121	3.81 × 10^–6^	0.10	0.005	0.026	*ASB18*, *GBX2*, *AGAP1*
	rs42882387	4	89,316,398	1.63 × 10^–6^	0.24	0.004	0.018	*POT1*
	rs137141507	6	3,362,250	1.32 × 10^–6^	0.09	0.006	0.018	*EXOSC9*, *CCNA2*, *BBS7*, *ANXA5*
	rs42487799	7	37,625,102	1.32 × 10^–9^	0.32	0.005	<0.001	*COMMD10*, *SEMA6A*
	rs109487947	7	54,827,253	1.07 × 10^–6^	0.08	−0.005	0.018	
	rs110233258	7	58,460,338	3.50 × 10^–8^	0.15	−0.005	0.001	*STK32A*, ***PPP2R2B***, *DPYSL3*
	rs109461455	9	37,700,129	1.92 × 10^–6^	0.15	−0.003	0.018	
	rs42864672	9	63,416,140	4.60 × 10^–8^	0.37	−0.004	0.001	
	rs136069526	9	67,796,936	1.04 × 10^–5^	0.48	−0.003	0.050	*ARHGAP18*
	rs109859987	11	53,890,637	9.83 × 10^–6^	0.49	−0.002	0.050	
	rs109992118	13	21,561,365	1.97 × 10^–6^	0.21	−0.003	0.018	*PLXDC2*
	rs109632400	13	58,602,292	1.73 × 10^–6^	0.35	−0.003	0.018	***PCK1***, *RBM38*, *RAE1*, *ZBP1*, ***CTCFL***, *PMEPA1*
	rs41751511	15	8,668,231	4.63 × 10^–7^	0.49	0.003	0.010	*CNTN5*
	rs43713533	15	28,453,282	2.87 × 10^–6^	0.29	−0.003	0.022	*TMPRSS13*, *FXYD2*, *FXYD6*
	rs42402130	16	32,852,344	2.86 × 10^–8^	0.23	0.005	0.001	*ADSS2*, *C16H1orf100*
	rs41750173	16	57,750,820	4.74 × 10^–6^	0.18	−0.004	0.029	*PAPPA2*
	rs29019796	17	25,645,239	4.84 × 10^–6^	0.05	0.007	0.029	
	rs110578750	18	2,907,599	1.05 × 10^–5^	0.14	−0.004	0.050	*GABARAPL2*, *CFDP2*, *CFDP1*, *TMEM170A*, *TMEM231*, *ADAT1*, *KARS1*, *TERF2IP*, *BCNT2*, *CHST6*
	rs110228250	20	40,015,842	3.52 × 10^–6^	0.05	−0.006	0.026	*ADAMTS12*, *SLC45A2*, ***RXFP3***
	rs41568642	21	12,166,884	2.40 × 10^–6^	0.17	0.004	0.020	
	rs110340891	22	37,375,611	9.41 × 10^–6^	0.19	0.003	0.050	*ATXN7*, *PSMD6*, *PRICKLE2*
NRR56	rs43587839	1	155,197,244	1.91 × 10^–7^	0.09	−0.003	0.003	
	rs134652568	2	74,743,823	4.21 × 10^–6^	0.15	0.003	0.024	
	rs110190075	2	103,750,400	7.13 × 10^–7^	0.28	0.005	0.009	
	rs43354413	3	92,138,083	7.02 × 10^–6^	0.30	0.002	0.036	*HSPB11*, *TMEM59*, *TCEANC2*, *MRPL37*, *SSBP3*, *LRRC42*, *CDCP2*, *CYB5RL*, *LDLRAD1*, *YIPF1*
	rs43371984	3	116,894,699	9.45 × 10^–6^	0.42	−0.004	0.043	*MLPH*, *LRRFIP1*, *PRLH*, *RAB17*, *COL6A3*
	rs43386888	4	49,509,741	2.41 × 10^–6^	0.12	−0.003	0.021	*NRCAM*, *NME8*, *PNPLA8*
	rs42766762	6	108,074,935	3.04 × 10^–6^	0.37	−0.005	0.021	
	rs110658771	8	19,488,876	3.48 × 10^–6^	0.17	0.003	0.022	*IZUMO3*
	rs109087862	8	63,729,696	7.17 × 10^–8^	0.46	−0.003	0.003	*ANKS6*, *GALNT12*, *GABBR2*
	rs110409952	9	87,035,097	2.88 × 10^–6^	0.39	0.002	0.021	*NUP43*, *PCMT1*, *LATS1*, *ULBP17*, *LRP11*, *KATNA1*, *ULBP21*
	rs133014180	10	66,613,339	1.56 × 10^–7^	0.11	0.004	0.003	***BMP4***
	rs41667346	11	23,497,697	5.49 × 10^–6^	0.33	−0.003	0.030	
	rs110387293	11	73,919,337	3.11 × 10^–7^	0.21	0.003	0.004	*POMC*, *DTNB*, *DNMT3A*
	rs41633184	13	13,837,771	4.25 × 10^–6^	0.09	0.005	0.024	
	rs43709749	14	28,478,462	1.08 × 10^–6^	0.44	0.002	0.011	
	rs41616446	15	13,400,749	9.97 × 10^–9^	0.26	−0.004	0.001	
	rs110815341	15	63,461,312	7.26 × 10^–6^	0.32	−0.003	0.036	*EIF3M*, *QSER1*, *PRRG4*, *DEPDC7*
	rs42427669	17	66,079,314	3.04 × 10^–7^	0.07	0.003	0.004	*SEZ6L*, *TPST2*, *SRRD*, *TFIP11*, *HPS4*, *CRYBB1*, *CRYBA4*, *ASPHD2*
	rs133424642	18	13,506,620	1.02 × 10^–6^	0.49	−0.003	0.011	*SLC7A5*, *CA5A*, *BANP*
	rs110863925	24	7,065,050	1.59 × 10^–7^	0.45	−0.003	0.003	*RTTN*, *SOCS6*
	rs135757150	24	54,887,194	3.08 × 10^–6^	0.45	0.003	0.021	*TCF4*
	rs109715869	25	25,154,075	7.91 × 10^–6^	0.44	0.002	0.037	*GSG1L*, *GTF3C1*, *KATNIP*, *IL21R*
	rs136986771	30	133,828,729	2.61 × 10^–6^	0.14	0.003	0.021	
	rs134555078	30	137,676,697	3.10 × 10^–8^	0.36	−0.004	0.002	
CE	rs29016910	2	41,127,106	4.83 × 10^–7^	0.42	0.001	0.007	*KCNJ3*
	rs43715311	3	114,365,299	5.28 × 10^–6^	0.44	0.001	0.034	*SH3BP4*
	rs43427376	5	7,184,325	1.41 × 10^–6^	0.28	0.001	0.015	*NAV3*
	rs133310180	5	90,520,626	2.21 × 10^–6^	0.35	0.001	0.019	*AEBP2*, *PLEKHA5*
	rs133412722	7	68,637,147	7.25 × 10^–6^	0.48	0.001	0.044	*HAVCR2*, *MED7*
	rs110471321	9	7,979,325	2.22 × 10^–7^	0.24	−0.001	0.005	*ADGRB3*
	rs108984322	10	8,009,471	2.40 × 10^–6^	0.18	−0.001	0.019	*IQGAP2*, ***F2RL2***, *F2R*, *S100Z*, ***CRHBP***, *AGGF1*, ***F2RL1***
	rs29017584	10	33,364,541	2.50 × 10^–6^	0.49	0.002	0.019	
	rs42568446	11	18,452,325	5.33 × 10^–7^	0.25	0.001	0.007	
	rs42583510	11	30,305,738	2.78 × 10^–6^	0.22	0.002	0.020	*MSH6*, *FBXO11*
	rs41628019	14	28,529,843	3.23 × 10^–7^	0.37	0.001	0.006	
	rs109928489	14	81,472,215	5.62 × 10^–7^	0.41	0.001	0.007	*COL14A1*, *DSCC1*, *DEPTOR*
	rs41824124	16	70,244,076	3.87 × 10^–6^	0.19	0.001	0.026	*RPS6KC1*
	rs41635371	16	78,603,871	1.52 × 10^–6^	0.40	−0.001	0.015	
	rs42813960	18	64,728,827	1.32 × 10^–7^	0.35	−0.001	0.004	*ZNF550*, *ZNF419*, *ZNF548*
	rs136257872	19	8,172,407	3.10 × 10^–10^	0.37	0.002	<0.001	*MSI2*
	rs108970271	22	26,765,132	5.86 × 10^–8^	0.30	−0.001	0.002	
	rs42070292	25	29,175,853	8.31 × 10^–10^	0.12	0.002	<0.001	*GALNT17*, *CALN1*
SB	rs41594258	1	34,978,818	1.06 × 10^–6^	0.09	−0.004	0.019	*VGLL3*
	rs109309140	2	4,981,764	1.42 × 10^–6^	0.46	0.002	0.022	*PROC*, *SFT2D3*, *WDR33*, *GPR17*, ***LIMS2***, *IWS1*, ***MYO7B***
	rs137754398	2	101,961,755	2.82 × 10^–9^	0.40	−0.005	<0.001	
	rs43353437	3	83,295,309	5.81 × 10^–6^	0.25	−0.003	0.039	*KANK4*, *DOCK7*, *USP1*
	rs43386788	4	27,127,668	3.98 × 10^–6^	0.09	−0.005	0.036	*HDAC9*
	rs43424011	5	7,320,087	6.04 × 10^–6^	0.31	−0.003	0.039	*NAV3*
	rs137802315	6	92,046,444	3.27 × 10^–6^	0.45	0.003	0.036	*CCNI*, *SEPTIN11*, ***CCNG2***
	rs41630520	9	19,561,418	6.43 × 10^–6^	0.23	−0.002	0.039	*TTK*, *ELOVL4*
	rs109624175	9	56,241,273	4.99 × 10^–8^	0.24	0.003	0.001	
	rs109920124	11	44,744,209	4.96 × 10^–6^	0.38	−0.003	0.039	*RANBP2*, *CCDC138*, *SULT1C3*, ***LIMS1***, *GCC2*, *EDAR*, *SULT1C4*, *SULT1C2*
	rs135416382	12	24,302,230	3.62 × 10^–6^	0.27	−0.003	0.036	*TRPC4*, *POSTN*
	rs134002839	13	71,259,389	3.58 × 10^–8^	0.18	−0.004	0.001	*PTPRT*
	rs135791311	14	71,330,255	1.44 × 10^–9^	0.09	0.006	<0.001	*TRIQK*
	rs43168517	16	10,976,232	5.53 × 10^–6^	0.10	0.004	0.039	
	rs108992403	19	16,143,462	4.99 × 10^–6^	0.20	−0.002	0.039	*ASIC2*
	rs134228482	22	13,014,212	3.13 × 10^–6^	0.41	−0.002	0.036	*MYRIP*, *EIF1B*
	rs41588424	28	12,833,134	3.37 × 10^–8^	0.17	−0.004	0.001	*CHRM3*
	rs42492371	29	1,952,181	1.88 × 10^–6^	0.50	−0.003	0.026	*FAT3*, *MTNR1B*
GL	rs42630203	1	18,608,496	1.18 × 10^–6^	0.30	−0.184	0.018	*CHODL*, *TMPRSS15*
	rs110604162	1	112,217,694	2.99 × 10^–6^	0.42	0.162	0.027	***PLCH1***
	rs43271952	1	134,240,312	4.60 × 10^–6^	0.30	0.174	0.033	*EPHB1*
	rs43354413	3	92,138,083	2.94 × 10^–6^	0.30	0.179	0.027	*HSPB11*, *TMEM59*, *TCEANC2*, *MRPL37*, ***SSBP3***, *LRRC42*, *CDCP2*, *CYB5RL*, *LDLRAD1*, *YIPF1*
	rs134191168	4	66,013,467	6.18 × 10^–8^	0.36	0.175	0.002	*ZNRF2*, *GGCT*, *NOD1*, *MTURN*
	rs108993952	7	48,852,799	1.00 × 10^–6^	0.39	−0.203	0.018	*SPOCK1*
	rs136460053	7	61,263,431	4.91 × 10^–6^	0.48	0.207	0.033	*HMGXB3*, *CSF1R*, *PPARGC1B*, *PDE6A*, *SLC26A2*
	rs109807989	8	88,740,000	1.92 × 10^–6^	0.28	−0.254	0.021	***CKS2***, *SECISBP2*, ***SEMA4D***, *SHC3*
	rs109727604	14	39,463,590	3.57 × 10^–6^	0.43	−0.155	0.028	
	rs136577145	16	30,791,818	1.73 × 10^–6^	0.37	−0.202	0.021	*TFB2M*, *CNST*, *SCCPDH*, *H3-5*, *SMYD3*
	rs41619483	18	6,140,925	3.40 × 10^–6^	0.29	0.152	0.028	***WWOX***
	rs110402487	18	64,444,876	2.27 × 10^–8^	0.29	−0.228	0.001	*AURKC*, *ZNF304*, *ZNF805*, *ZNF548*, *ZIM3*
	rs109173977	21	41,990,869	3.80 × 10^–7^	0.35	−0.328	0.010	*GPR33*, *HEATR5A*, *NUBPL*
	rs110148531	23	39,148,251	1.09 × 10^–6^	0.19	−0.175	0.018	*RNF144B*
	rs137469593	27	19,456,244	1.74 × 10^–6^	0.37	−0.170	0.021	*PDGFRL*, *PCM1*, *FGL1*, ***MTUS1***
	rs135655219	28	5,171,795	1.79 × 10^–8^	0.26	0.236	0.001	*SIPA1L2*
	rs42178394	29	33,061,591	7.27 × 10^–6^	0.17	−0.168	0.047	*JAM3*, *IGSF9B*, *SPATA19*

*CR, conception rate; NRR56, 56-day non-return rate; CE, calving ease; SB, stillbirth; GL, gestation length; MAF, minor allele frequency. ^a^The important candidate genes for each trait are shown in bold face.*

**TABLE 5:** Significant single-nucleotide polymorphism (SNP) and near-by genes of the genetic effects of dam on five reproductive traits carried out by genome-wide association studies in Holstein cattle.

Trait	SNP	BTA	Position (bp)	*P*-value	MAF	Effect	FDR	Genes^a^
CR	rs110588394	1	157,046,373	8.55 × 10^–9^	0.19	0.008	0.001	*PP2D1*, *EFHB*, *RAB5A*
	rs135745940	2	86,206,807	5.58 × 10^–8^	0.23	0.006	0.002	*MARS2*, *COQ10B*, *PLCL1*, *RFTN2*, *HSPD1*, *HSPE1*, *MOB4*, *BOLL*
	rs41625668	3	2,131,858	7.13 × 10^–7^	0.44	0.005	0.010	*TADA1*, *POGK, ILDR2*
	rs110992111	3	63,079,015	1.70 × 10^–6^	0.45	−0.004	0.017	*ADGRL2*
	rs41657989	7	24,218,169	1.10 × 10^–7^	0.40	−0.005	0.003	*CHSY3*, *ADAMTS19*, *MINAR2*
	rs41592654	9	32,237,919	1.38 × 10^–7^	0.21	−0.005	0.003	*ASF1A*, *MCM9*, *CEP85L*, *PLN*, *FAM184A*
	rs42557707	12	45,131,104	5.35 × 10^–7^	0.08	−0.008	0.008	
	rs110113735	12	75,616,202	2.53 × 10^–6^	0.17	0.004	0.021	*FARP1, SLC15A1*, *DOCK9, STK24*
	rs137128437	14	73,073,273	5.01 × 10^–7^	0.41	−0.005	0.008	*SLC26A7*
	rs43713566	15	29,560,389	9.42 × 10^–7^	0.20	0.006	0.011	*BCL9L*, *VPS11*, *HMBS*, *DPAGT1*, *TRAPPC4*, *SLC37A4*, *DDX6*, *HYOU1*, *ABCG4*, *UPK2*, *FOXR1*, *C2CD2L*, *HINFP*, *CENATAC*, *RPS25*, *NLRX1*, *CXCR5*
	rs136206713	15	81,820,622	2.82 × 10^–8^	0.41	0.006	0.002	*CNTF*, *LPXN*
	rs132777210	21	69,731,178	2.35 × 10^–6^	0.34	0.005	0.021	*BTBD6*, *BRF1*, *TMEM121*, *PACS2*, *TEDC1*, *CRIP1*, *NUDT14*
	rs109776480	22	57,040,932	1.13 × 10^–6^	0.43	−0.005	0.012	*MRPS25*, *RBSN*, *SYN2*, *TIMP4*
NRR56	rs42827552	6	23,199,236	3.74 × 10^–6^	0.35	−0.005	0.041	*BANK1*
	rs41575824	9	53,639,845	3.48 × 10^–6^	0.12	0.007	0.041	*FUT9*
	rs136204465	14	9,422,558	2.62 × 10^–7^	0.15	−0.005	0.005	
	rs134979761	17	9,110,531	2.75 × 10^–8^	0.41	0.006	0.002	
	rs109533406	17	57,716,043	2.82 × 10^–7^	0.39	0.006	0.005	*NOS1*, *FBXO21*, *KSR2*
	rs135974611	18	31,152,142	2.34 × 10^–6^	0.27	−0.006	0.032	
	rs41606596	18	45,936,015	1.35 × 10^–7^	0.07	−0.006	0.004	*FAM187B*, *GRAMD1A*, *SCN1B*, *HPN*, *FFAR2*, *CD22*, *FFAR3*, *LSR*, *USF2*, *HAMP*, *MAG*, *LGI4*, *FXYD1*, *FXYD7*, *FXYD5*, *FFAR1*
	rs110401168	19	57,629,224	3.60 × 10^–9^	0.35	0.008	<0.001	
	rs42428874	21	36,656,375	4.58 × 10^–6^	0.19	−0.005	0.042	*NOVA1*
	rs136876790	22	34,075,088	4.90 × 10^–8^	0.47	0.005	0.002	*SUCLG2*
	rs110534364	24	50,494,672	4.31 × 10^–6^	0.11	0.007	0.042	*ELAC1*, ***SMAD4***, *MEX3C*, *ME2*, *MRO*
	rs109783875	26	27,091,833	1.71 × 10^–6^	0.36	−0.004	0.027	
CE	rs110752117	7	51,418,311	1.21 × 10^–6^	0.08	−0.007	0.019	*PURA*, *NRG2*, ***CYSTM1***, ***HBEGF***, *SLC4A9*, *IGIP*, *PFDN1*
	rs110761813	10	16,708,640	1.63 × 10^–6^	0.37	−0.005	0.022	
	rs109942798	12	18,661,493	1.07 × 10^–6^	0.08	0.005	0.019	*MLNR*, *FNDC3A*
	rs109156982	13	47,716,129	6.75 × 10^–8^	0.28	0.005	0.002	*GPCPD1*, *PROKR2*
	rs110115548	13	67,003,397	3.93 × 10^–6^	0.20	0.005	0.048	*TTI1*, *VSTM2L*, *RPRD1B*, *BPI*, ***TGM2***, *KIAA1755*
	rs109493014	15	72,384,357	1.12 × 10^–9^	0.43	0.005	<0.001	
	rs41578821	16	30,855,245	9.26 × 10^–8^	0.10	−0.005	0.003	***TFB2M***, *CNST*, *H3-5*, ***SMYD3***
	rs42949634	18	39,542,279	4.32 × 10^–7^	0.25	0.004	0.009	*CALB2*, *TAT*, *AP1G1*, *MARVELD3*, *PHLPP2*, *CHST4*, *ZNF19*, *ZNF23*
	rs42534666	26	5,084,073	6.45 × 10^–9^	0.14	0.008	<0.001	*PCDH15*
SB	rs135087719	4	69,282,296	4.09 × 10^–7^	0.18	−0.001	0.004	*SKAP2*
	rs135473218	7	95,890,433	8.29 × 10^–7^	0.23	0.001	0.008	*CAST*, *PCSK1*
	rs43546352	8	31,779,503	1.34 × 10^–10^	0.25	−0.002	<0.001	*TYRP1*
	rs134655277	9	4,579,294	1.88 × 10^–6^	0.45	0.001	0.015	
	rs135323642	9	67,192,772	2.79 × 10^–6^	0.38	0.002	0.020	*LAMA2*
	rs41595401	10	38,651,279	9.51 × 10^–7^	0.33	0.001	0.008	
	rs110003547	11	47,431,717	4.84 × 10^–9^	0.07	0.001	<0.001	*EIF2AK3*, *RPIA*, *TEX37*, *FOXI3*
	rs42337856	11	58,828,979	2.61 × 10^–7^	0.27	−0.001	0.003	
	rs133162533	15	3,989,919	5.61 × 10^–6^	0.48	0.001	0.036	
	rs41790653	16	9,685,690	1.47 × 10^–7^	0.47	0.001	0.003	
	rs133390427	17	60,309,111	3.61 × 10^–6^	0.33	0.001	0.025	*TBX5*
	rs110008365	18	5,593,593	2.74 × 10^–7^	0.28	−0.001	0.003	***WWOX***
	rs109395549	20	523,765	4.10 × 10^–7^	0.42	0.001	0.004	*PANK3*, *SLIT3*
	rs110695662	21	44,838,064	1.22 × 10^–9^	0.17	0.001	<0.001	*EAPP*, *SNX6*, *SPTSSA*
	rs42566616	22	40,247,823	1.65 × 10^–10^	0.36	0.001	<0.001	
	rs137494875	22	47,178,548	1.88 × 10^–7^	0.21	−0.001	0.003	*CACNA1D*, *CHDH*, *IL17RB*, *ACTR8*, *SELENOK*
	rs109564594	26	1,419,659	8.41 × 10^–8^	0.20	−0.001	0.002	
GL	rs43747887	1	88,621,323	2.52 × 10^–6^	0.49	0.183	0.031	
	rs135396670	2	130,043,555	2.25 × 10^–6^	0.32	−0.172	0.031	*C1QA*, *C1QC*, ***C1QB***, *EPHA8*, *LACTBL1*, ***EPHB2***, *TEX46*
	rs42760413	6	90,933,892	5.91 × 10^–7^	0.17	−0.168	0.013	***CXCL10***, *CXCL11*, *SDAD1*, *ART3*, *NUP54*, *SCARB2*, *PPEF2*, *NAAA*, *CXCL9*
	rs43482393	6	93,053,914	1.09 × 10^–9^	0.34	−0.349	<0.001	*FRAS1*
	rs109176316	7	52,159,740	1.67 × 10^–6^	0.31	−0.175	0.026	***PCDHA13***, *PCDHB1*, *PCDHAC2*
	rs136804356	14	28,876,699	4.75 × 10^–6^	0.33	0.158	0.047	
	rs136577145	16	30,791,818	3.97 × 10^–10^	0.39	−0.244	<0.001	*TFB2M*, *CNST*, *SCCPDH*, *H3-5*, *SMYD3*
	rs109102279	20	19,138,824	6.01 × 10^–8^	0.12	0.250	0.002	
	rs42406702	20	55,193,435	3.00 × 10^–6^	0.12	−0.229	0.033	
	rs110072536	25	40,344,012	8.43 × 10^–7^	0.31	−0.170	0.015	*CARD11*
	rs109960049	26	17,360,622	4.05 × 10^–8^	0.48	−0.326	0.001	*ENTPD1*, *ZNF518A*, *BLNK*, *CCNJ*

*CR, conception rate; NRR56, 56-day non-return rate; CE, calving ease; SB, stillbirth; GL, gestation length; MAF, minor allele frequency. ^a^The important candidate genes for each trait are shown in bold face.*

**TABLE 6:** Significant single-nucleotide polymorphisms (SNPs) of the genetic effects of service sire and overlapping quantitative trait loci (QTLs).

Trait	SNP	BTA	QTL^a^
CR	rs109461455	9	Muscle anserine content QTL (151506)
	rs109632400	13	Interval to first estrus after calving QTL (14769)
	rs43713533	15	Body weight (yearling) QTL (68806)
	rs110228250	20	Milk protein percentage QTL (105842)
NRR56	rs41616446	15	Body weight (yearling) QTL (68771)
	rs42427669	17	Conception rate QTL (177207)
CE	rs43715311	3	Milking speed QTL (157395)
	rs43427376	5	Milk unglycosylated kappa-casein percentage QTL (119033)
	rs108984322	10	Hip height QTL (131441); udder depth QTL (135447)
SB	rs41594258	1	Milk protein yield QTL (26122)
	rs137802315	6	Milk unglycosylated kappa-casein percentage QTL (118721); milk kappa-casein percentage QTL (111024)
GL	rs108993952	7	Milking speed QTL (157567); body depth QTL (43024); calving ease (maternal) QTL (43025); daughter pregnancy rate QTL (43026); foot angle QTL (43027); milk fat percentage QTL (43028); PTA type QTL (43029); udder attachment QTL (43030); milk fat yield QTL (43031); net merit QTL (43032); length of productive life QTL (43033); rump width QTL (43034); calving ease QTL (43035); somatic cell score QTL (43036); stillbirth QTL (43037); stature QTL (43038); strength QTL (43039); udder depth QTL (43040)

*CE, calving ease; SB, stillbirth; GL, gestation length. ^a^The QTL mapping was based on the Cattle QTL Database (https://www.animalgenome.org/cgi-bin/QTLdb/BT/index), and those QTLs that include significant SNPs are shown in the table.*

**TABLE 7:** Significant single-nucleotide polymorphisms (SNPs) of the genetic effects of dam and overlapping quantitative trait loci (QTLs).

Trait	SNP	BTA	QTL^a^
CR	rs41657989	7	Milking speed QTL (157466); clinical mastitis QTL (19014)
NRR56	rs109533406	17	Muscle potassium content QTL (152009)
CE	rs110115548	13	Milk yield QTL (16180); milk protein yield QTL (16181); milk fat percentage QTL (16182); milk protein percentage QTL (16183)
	rs41578821	16	Body weight (slaughter) QTL (102172)
SB	rs41595401	10	Body weight (yearling) QTL (68167); body weight gain QTL (68168); body depth QTL (44657); dairy form QTL (44658); feet and leg conformation QTL (44659); PTA type QTL (44660); teat placement—front QTL (44661); udder attachment QTL (44662); net merit QTL (44663); teat placement—rear QTL (44664); udder height QTL (44665); rump width QTL (44666); somatic cell score QTL (44667); stature QTL (44668); strength QTL (44669); udder cleft QTL (44670); udder depth QTL (44671)
	rs110003547	11	Age at puberty QTL (21140)
	rs42337856	11	Interval to first estrus after calving QTL (28582)
	rs109564594	26	Calving ease (maternal) QTL (52571); dairy form QTL (52572); daughter pregnancy rate QTL (52573); milk fat percentage QTL (52574); milk fat yield QTL (52575); net merit QTL (52576); length of productive life QTL (52577); milk protein percentage QTL (52578); milk protein yield QTL (52579); rump angle QTL (52580); rear leg placement—side view QTL (52581); teat placement—rear QTL (52582); calving ease QTL (52583); teat length QTL (52584); udder cleft QTL (52585)

*CR, conception rate; NRR56, 56-day non-return rate; CE, calving ease; SB, stillbirth; GL, gestation length. ^a^The QTL mapping was based on the Cattle QTL Database (https://www.animalgenome.org/cgi-bin/QTLdb/BT/index), and those QTLs that include significant SNPs are shown in the table.*

For GESS, 100 SNPs were found to be significant, with 23, 24, 18, 18, and 17 associated with CR, NRR56, CE, SB, and GL, respectively (Table 4). For the success traits, 53 nearby annotated protein-coding genes located 200 kb upstream and downstream of the significant SNPs were mapped, including genes related to sperm development (e.g., *BMP4*) and early embryogenesis (e.g., *LRRC34*). Han and Peñagaricano (2016) also identified a genomic region (1.5 Mb) located on BTA13 which explained more than 0.50% of the total additive genetic variance for SCR (male fertility). This region contains the *CTCFL* gene detected in the present study. Although previous studies have attempted to identify candidate genes related to GESS on success traits (Taylor et al., 2018), novel candidate genes were identified in this study—for instance, the gene *BMP4* (bone morphogenetic protein 4) was previously indicated as a candidate gene for spermatogenesis (Hu et al., 2004; Li et al., 2014) and follicle development (Nilsson and Skinner, 2003; Shimizu et al., 2004; Fatehi et al., 2005; Li and Ge, 2011). These associations indicate the paternal and maternal effects on the pre-implantation stage of embryo development, respectively (Koide et al., 2009; Li et al., 2012a). In this context, the present study also revealed the GESS on female conception. Ou et al. (2020) carried out a transcriptome analysis on spermatogonial stem cells (SSCs) and reported that *LRRC34* was highly expressed in mouse SSCs and was essential for *in vitro* SSC proliferation. *RXFP3* was identified to be differentially expressed in human sperm and was likely diminished in spermiogenesis (Heidari et al., 2018). Furthermore, other studies suggested *PPP2R2B* and *PCK1* as candidate genes affecting the semen quality traits in livestock (Huang et al., 2016; Gao et al., 2019).

One hundred fourteen nearby proteinase genes located 200 kb upstream and downstream of the significant SNPs related to the GESS of calving traits were mapped. The fetal growth and metabolism show a specific pattern during pregnancy (Bell et al., 1993) and supposedly reflect on calving traits. Some potential genes related to calving traits in our study were previously associated with carcass and meat quality traits, including *MTUS1* (Albrecht et al., 2016), *PLCH1* (Lemos et al., 2016), *F2RL1* (Srikanth et al., 2020; Zhang et al., 2017), *MYO7B* (Doyle et al., 2020; Jia et al., 2020), *WWOX* (Grigoletto et al., 2020), *TFB2M* (Jiang et al., 2006; Song et al., 2019), and *SMYD3* (De Vos, 2018). Furthermore, *F2RL1* was reported to affect the body size in Chinese Holstein cattle (Zhang et al., 2017). Some other genes were identified as essential genes for embryogenesis [*SEMA4D* (Masuda et al., 2004), and *CCNG2* (Ma et al., 2015) and *CKS2* (Martinsson-Ahlzén et al., 2008), which contribute to subsequent fetal development]. Perkins et al. (1995) collected human fetal plasma samples both at mid-gestation and parturient to explore the trend of corticotrophin-releasing hormone-binding protein (CRHBP) during the different gestation stages and reported CRHBP as being functional in both maternal and fetal circulation. *F2RL2*, *LIMS2*, and *LIMS1* were indicated by Forde et al. (2012) as pregnancy-associated genes that are differently expressed in the endometrium of cattle during early pregnancy, indicating the potential role of these genes on successful pregnancy establishment. The SNP *rs43354413* located in BTA 3 was identified as significant for GESS on NRR56 and GL, with 10 protein-coding genes in close proximity—for instance, the *SSBP3* gene (located approximately 131 kb upstream of this marker) was reported to regulate mouse embryonic stem cell differentiation to trophoblast-like cells (Liu J. et al., 2016). These findings suggest the possible function of *SSPB3* of GESS on reproduction performance. We used Chinese Holstein population and re-defined the genetic components of calving traits in current study, but some SNPs (*rs42813960* and *rs110402487*) in our study are consistent with previous genomic studies of calving traits focused on BTA18 (Müller et al., 2017), indicating the potential importance of this chromosome in calving performance. Fang et al. (2019) proposed *ZNF613* as a candidate gene for paternal contributions to GL, and in our population, we detected one GL-related marker located downstream of *ZNF613* (*rs110402487*). Furthermore, we also mapped these SNPs to the Animal QTL Database (see Text Footnote 3) and subsequently found that some of them were located in QTLs associated with related traits (Table 6). Particularly, *rs108993952* was related to GESS on GL and meanwhile located in the QTL related to some maternal calving traits, which is a promising candidate marker for calving performance.

**FIGURE 5:**
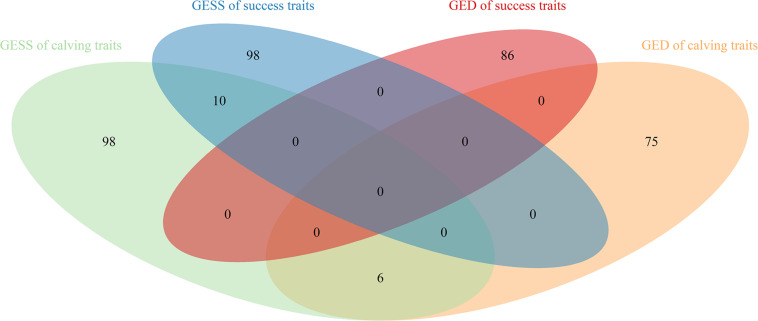
Venn diagram of genes identified for the genetic effect of service sire and genetic effect of dam on success and calving traits in Holstein cattle.

Sixty-two SNPs were found to be significant for GED, with 13, 12, 9, 17, and 11 being associated with CR, NRR56, CE, SB, and GL, respectively (Table 5). One hundred sixty-seven protein-coding genes were found within 200 kb of those SNPs. *rs136577145* was significant for both GESS and GED on GL and was about 63 kb upstream of *rs41578821*, which was detected as significant for GED on CE. The nearby genes mapped with *rs136577145* were *TFB2M*, *CNST*, *SCCPDH*, *H3-5*, and *SMYD3*. *TFB2M*, a mitochondrial transcription specificity factor, as well as *SMYD3*, a histone lysine methyltransferase, were previously linked to bone and skeletal muscle tissue development (Norrbom et al., 2010; Fujii et al., 2011; Sun et al., 2015)—that is, both paternal- and maternal-derived effects on GL were coincident with growth ability. In addition, *WWOX* overlapped between GESS and GED on other calving traits, though no gene overlapped between GESS and GED on success traits (Figure 5). This is consistent with the genetic correlation estimates observed (Table 2). The low overlap between GESS and GED on success traits demonstrates that different genes are involved in the insemination outcomes from the dam and service sire contributions.

Except for the genes mentioned before, the genomic results of GED are consistent with previous studies (e.g., *SMAD4*). For GED on success traits, 86 nearby protein-coding genes located 200 kb upstream and downstream of significant SNPs were mapped. Particularly, *SMAD4* has been reported to be linked to embryogenesis and folliculogenesis in mice (Chu et al., 2004; Pangas et al., 2006). Lee et al. (2014) reported *SMAD4* to be involved in early embryonic development in cattle and to regulate the effects of follistatin, which influences the environment-independent part of the interval from the first to the last insemination in cattle (Zhang et al., 2019). Eighty-one protein-coding genes were mapped for GED on calving traits and previously considered as essential genes for the maternal mechanism during pregnancy, including *CXCL10* (Walker et al., 2012), *HBEGF* (Jessmon et al., 2009), *PCDHA13* (Lotfan et al., 2018), *TGM2* (Purfield et al., 2019), *C1QB* (Cochran et al., 2013), *CYSTM1* (Purfield et al., 2019), and *EPHB2* (Purfield et al., 2015), which were also identified as candidate genes for maternal effect on calving traits in dairy and beef cattle. We also observed that some significant SNPs located in QTLs are associated with related traits (Table 7)—for instance, *rs42427669*, which was related to GED on NRR56, is located in a QTL region associated with CR (Kiser et al., 2019), while the GL-related marker *rs108993952* is located in QTLs associated with CE and SB (Cole et al., 2011).

#### Functional Enrichment Analyses

The enriched GO terms and KEGG pathways that passed the criteria of the Benjamini–Hochberg-corrected *p*-value < 0.05 are summarized in the Supplementary Files, and the genes shared between the main terms or pathways are presented in Supplementary Figures 2–5. Noticeably, both GESS and GED on success traits were enriched in neural development-related terms, such as neural crest cell migration (GO:0001755) for GESS on success traits and positive regulation of neuron projection development (GO:0010976) for GED on success traits. For GESS on calving traits, genes were enriched mainly in the thrombin-activated receptor signaling pathway (GO:0070493), microvillus (GO:0005902), neural precursor cell proliferation (GO:0061351), liver development (GO:0001889), *N*-methyltransferase activity (GO:0008170), sulfotransferase activity (GO:0008146), and cyclin-dependent protein kinase holoenzyme complex (GO:0000307). These categories include functionable genes named *CCNG2* and *CKS2* (GO:0000307), *LIMS2* (GO:0001889 and GO:0061351), *MYO7B*, *WWOX* (GO:0005902), *SMYD3*, *TFB2M* (GO:0008170), *F2RL1*, and *F2RL2* (GO:0070493) as previously discussed. In addition, the enrichment analysis for GED showed main terms such as *CXCR3* chemokine receptor binding (GO:0048248), homophilic cell adhesion via plasma membrane adhesion molecules (GO:0007156), and synapse pruning (GO:0098883), including *CXCL10* (GO:0048248), *PCDHA13* (GO:0007156), and *C1QB* (GO:0098883). Therefore, our findings provide further evidence of the possible genetic mechanism of GESS and GED on reproductive performance in Holstein cattle.

### Future Prospects

Generally, only the GED for reproduction performance is analyzed in genetic evaluations, though research including the current study have shown the need to dissect GESS (Tiezzi et al., 2011). Compared to previous studies, we fitted an improved model considering the covariance between GESS and GED and also identified candidate genes associated with GESS and GED. The GESS on reproductive traits is small but significant. The low repeatability estimates indicate the poor consistency among repeated records. Therefore, more accurate records and novel traits are required. With recent improvements in data collection, GESS might become an important factor on the genetic evaluation of reproductive performance. Genomic selection is also expected to contribute to improve the accuracy of breeding value for these lowly heritable traits (Rice and Lipka, 2019). Additional analyses with larger datasets and in independent populations (e.g., different breeds) are recommended.

## Conclusion

The GESS on reproductive traits is heritable, with a similar genetic variance to the GED. Moreover, the approximate genetic correlation among the GESS and production, health, and type traits is unfavorable for the success traits (CR and NRR56) but favorable for the calving traits (CE, SB, and GL). A total of 100 and 62 significant SNPs were detected to be associated with GESS and GED on those five reproductive traits, respectively. Among them, five genes (*BMP4* and *CTCFL* for success traits and *WWOX*, *TFB2M*, and *SMYD3* for calving traits) are suggested as important candidate genes for GESS according to positional and functional analyses. As GESS and GED are lowly heritable, genomic prediction might be a promising alternative for breeding schemes aiming to improve fertility performance in dairy cattle.

## Data Availability Statement

The data analyzed in this study is subject to the following licenses/restrictions: this manuscript utilizes proprietary data. Requests to access these datasets should be directed to YW, wangyachun@cau.edu.cn.

## Ethics Statement

The studies involving animals were reviewed and approved by the Animal Welfare Committee of the China Agricultural University (Protocol Number: DK996).

## Author Contributions

ZC and YW conceived the study. ZC and LB designed the analyses, discussed the results, and drafted the manuscript. ZC performed all the data analyses and the data curation of genotype. ZC, HL, and RS prepared the phenotypic files. YC assisted with the modeling analyses. LL and GG provided support for the collection of raw data. All authors have read and approved the final version of the manuscript.

## Conflict of Interest

LL is employed by Beijing Dairy Cattle Center. GG is employed by Beijing Sunlon Livestock Development Company Limited. The remaining authors declare that the research was conducted in the absence of any commercial or financial relationships that could be construed as a potential conflict of interest.

## Publisher’s Note

All claims expressed in this article are solely those of the authors and do not necessarily represent those of their affiliated organizations, or those of the publisher, the editors and the reviewers. Any product that may be evaluated in this article, or claim that may be made by its manufacturer, is not guaranteed or endorsed by the publisher.

## Footnotes

1
www.afimilk.com.cn

2
http://genome.ucsc.edu/cgi-bin/hgLiftOver

3
www.animalgenome.org/cgi-bin/QTLdb/BT/index

## Supplementary Material

Supplementary Figure 1 The linkage disequilibrium decay of the population.

Supplementary Figure 2 The Gene Ontology- and Kyoto Encyclopedia of Genes and Genomes-based network analysis of candidate genes of genetic effect of service sire on success traits using ClueGO application in Cytoscape.

Supplementary Figure 3 The Gene Ontology- and Kyoto Encyclopedia of Genes and Genomes-based network analysis of candidate genes of genetic effect of service sire on calving traits using ClueGO application in Cytoscape.

Supplementary Figure 4 The Gene Ontology- and Kyoto Encyclopedia of Genes and Genomes-based network analysis of candidate genes of genetic effects of the dam on success traits using ClueGO application in Cytoscape.

Supplementary Figure 5 The Gene Ontology- and Kyoto Encyclopedia of Genes and Genomes-based network analysis of candidate genes of genetic effects of the dam on calving traits using ClueGO application in Cytoscape.

## Abbreviations

GED: genetic effects of the dam
SCR: sire conception rate
GESS: genetic effect of service sire
GWAS: genome-wide association study
CR: conception rate
NRR56: 56-day non-return rate
CE: calving ease
SB: stillbirth
GL: gestation length
SNP: single-nucleotide polymorphism
MAF: minor allele frequency
FDR: false discovery rate
QTL: quantitative trait locus.
