# Supplementary material for: Genetic and Genomic Analyses of Service Sire Effect on Female Reproductive Traits in Holstein Cattle
Source: Front Genet. 2021 Sep 3;12:713575. doi: 10.3389/fgene.2021.713575 (PMC8446201; doi:10.3389/fgene.2021.713575)
Supplement: Supplementary Figure 1 — The linkage disequilibrium decay of the population. [file Data_Sheet_1.docx]

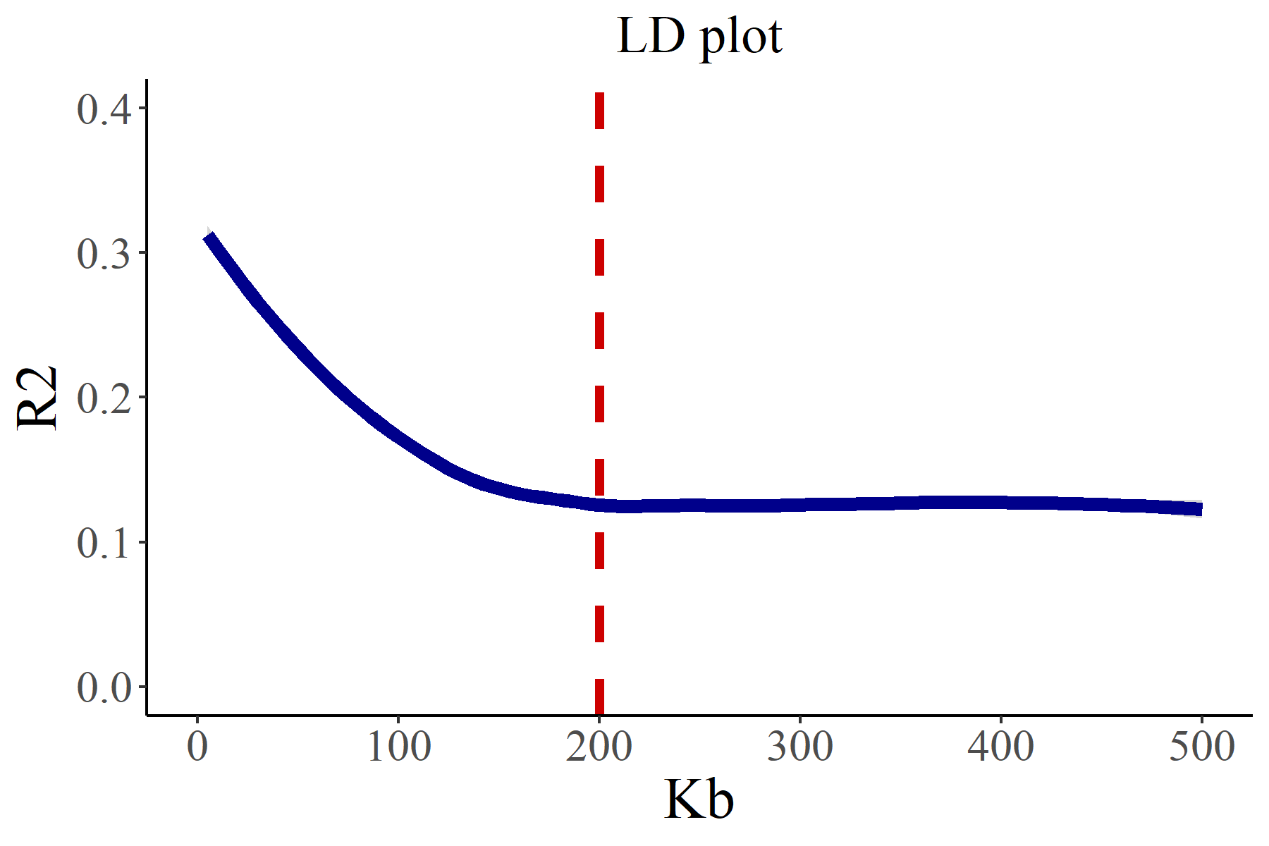


**Figure S1.** The linkage disequilibrium (LD) decay of the population. The x-axis and y-axis were distance between adjacent SNP and their average square of correlation coefficient (r^2^). 200 kb was chosen as the LD decay distance (dark red dotted line).


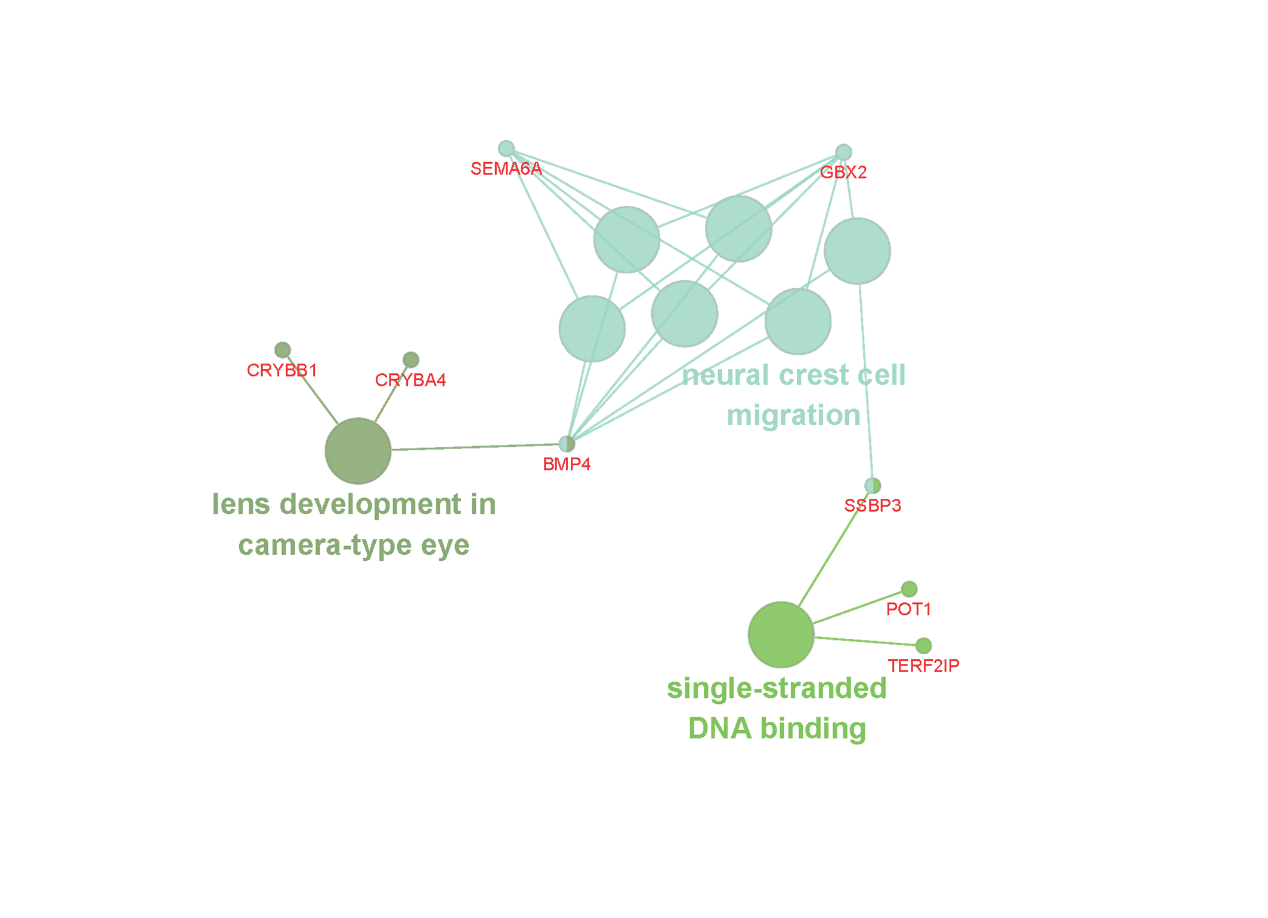


**Figure S2.** The GO-and-KEGG-based network analysis of candidate genes of GESS on success traits using ClueGO application in Cytoscape. Main terms and pathways with Benjamini-Hochberg-corrected P-value < 0.05 and genes who shared between terms and pathways were shown.


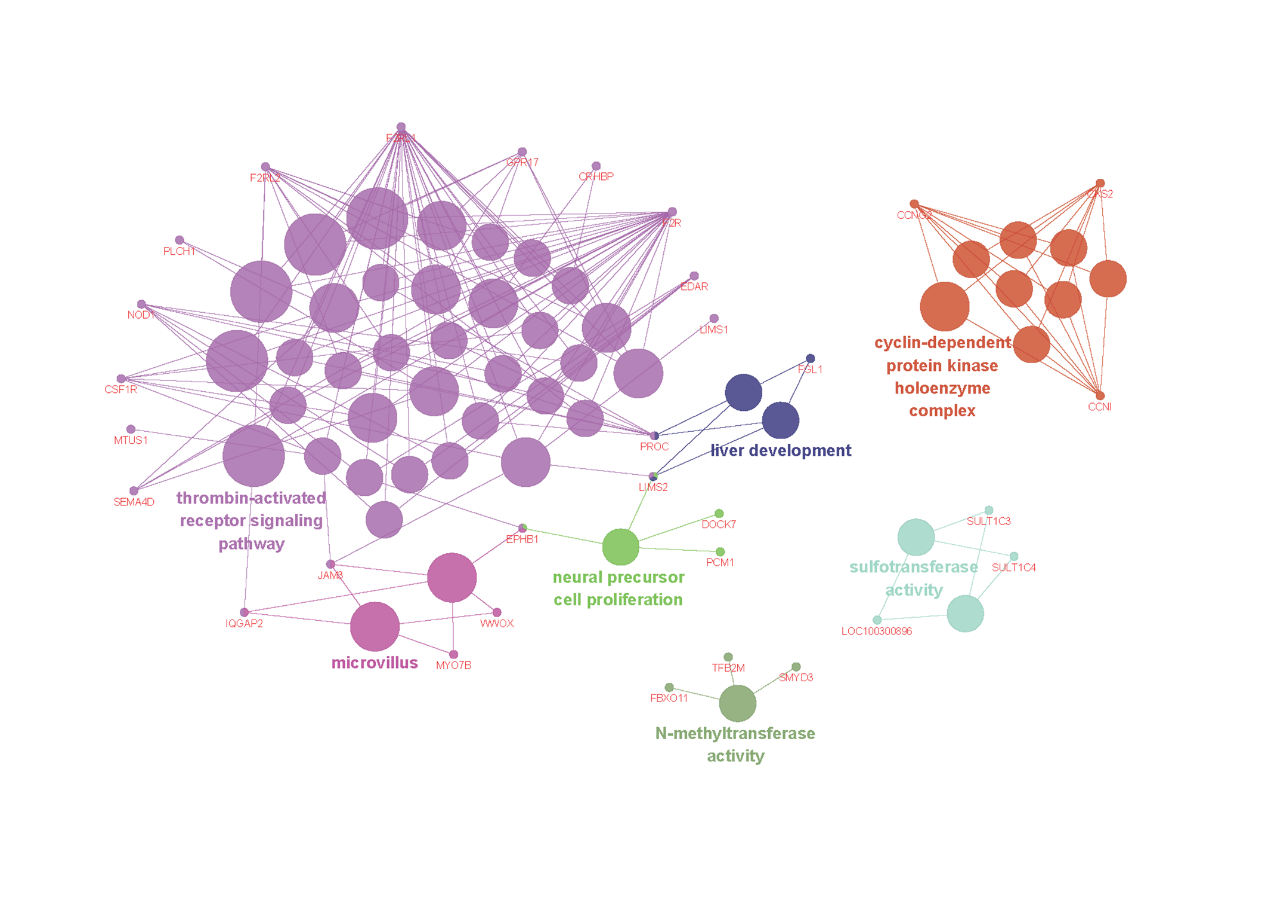


**Figure S3.** The GO-and-KEGG-based network analysis of candidate genes of GESS on calving traits using ClueGO application in Cytoscape. Main terms and pathways with Benjamini-Hochberg-corrected P-value < 0.05 and genes who shared between terms and pathways were shown.


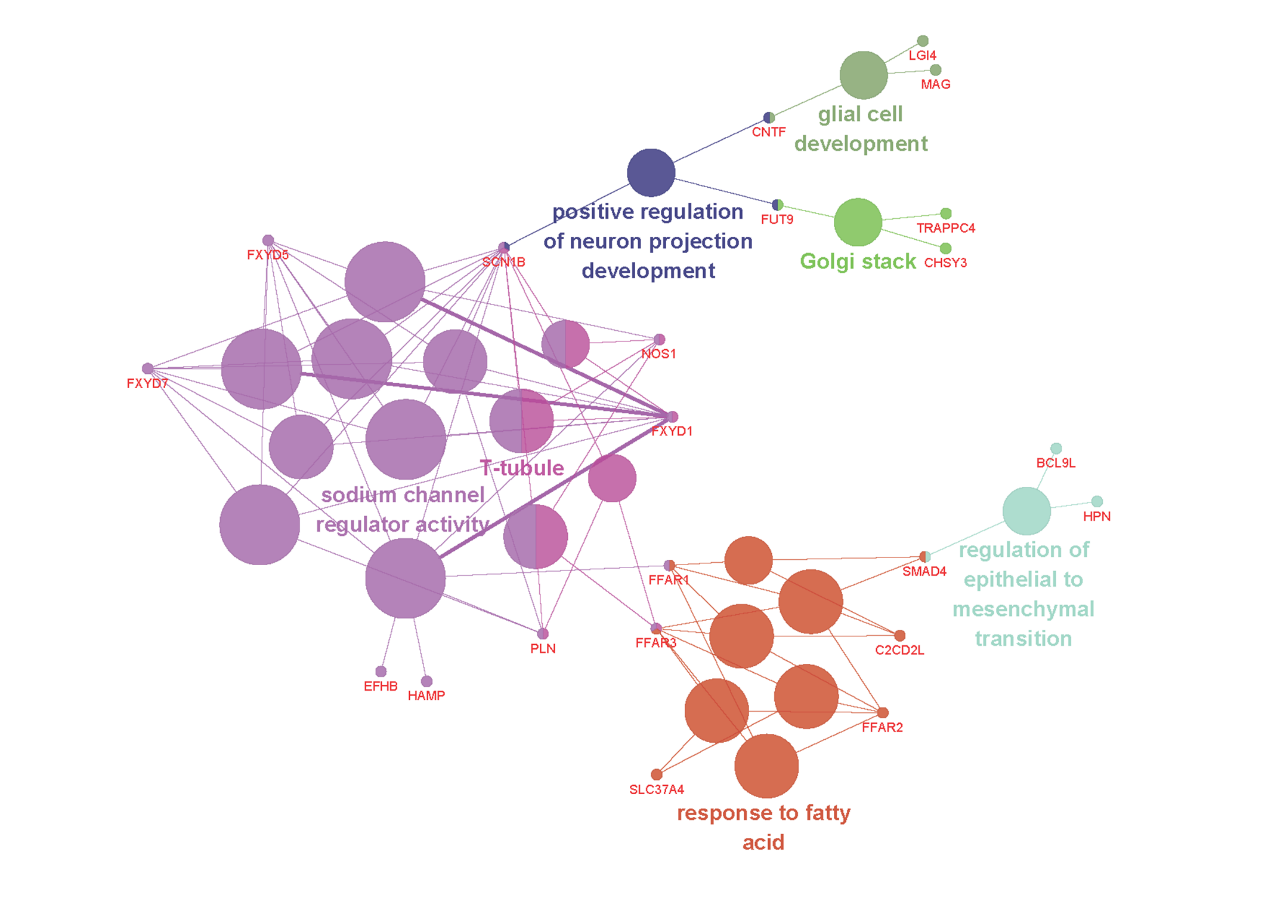


**Figure S4.** The GO-and-KEGG-based network analysis of candidate genes of GED on success traits using ClueGO application in Cytoscape. Main terms and pathways with Benjamini-Hochberg-corrected P-value < 0.05 and genes who shared between terms and pathways were shown.


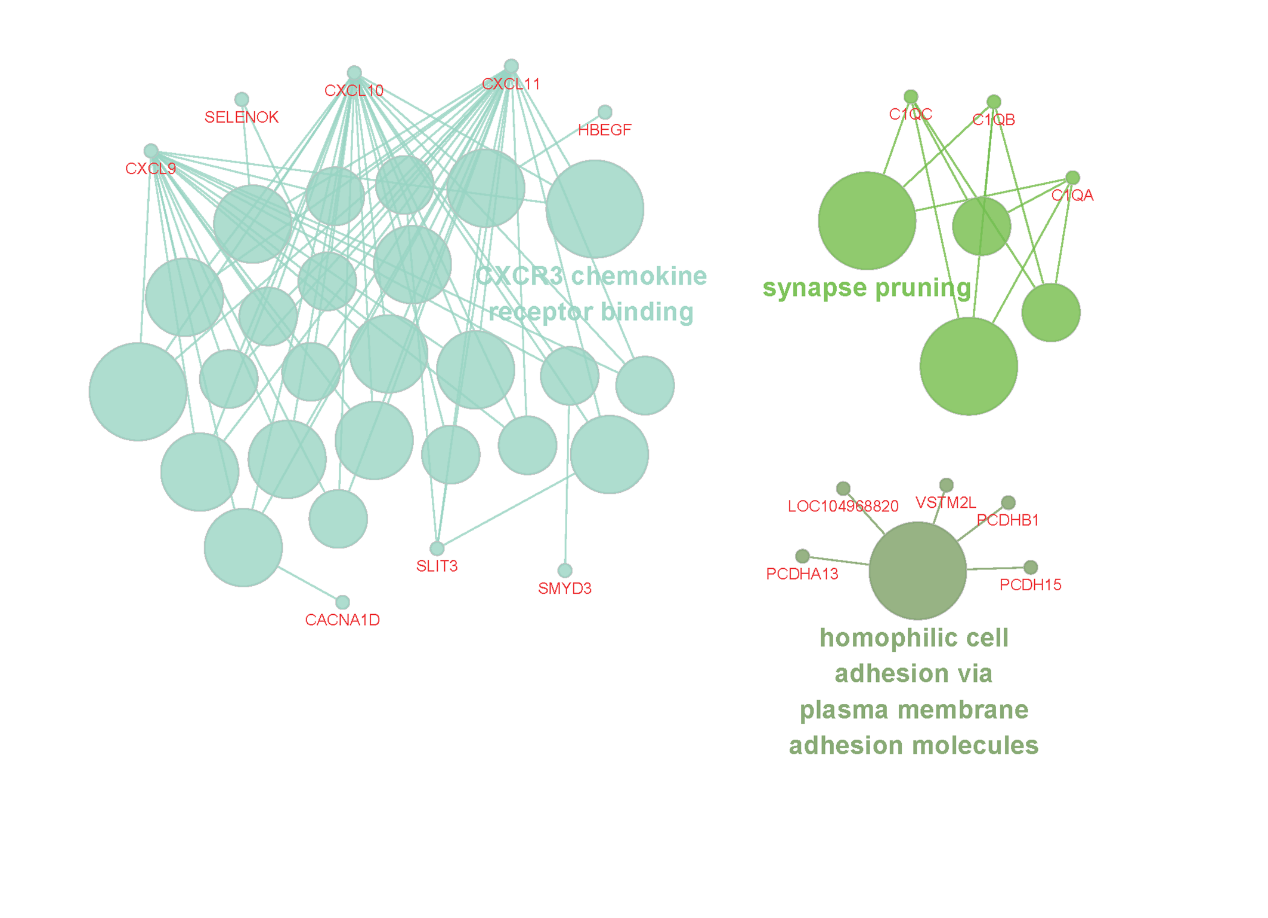


**Figure S5.** The GO-and-KEGG-based network analysis of candidate genes of GED on calving traits using ClueGO application in Cytoscape. Main terms and pathways with Benjamini-Hochberg-corrected P-value < 0.05 and genes who shared between terms and pathways were shown.
